# Estimating and characterizing the burden of multimorbidity in the community: A comprehensive multistep analysis of two large nationwide representative surveys in France

**DOI:** 10.1371/journal.pmed.1003584

**Published:** 2021-04-26

**Authors:** Joël Coste, José M. Valderas, Laure Carcaillon-Bentata

**Affiliations:** 1 Public Health France, Saint-Maurice, France; 2 APEx Collaboration for Academic Primary Care, Health Services and Policy Research Group, University of Exeter, Exeter, United Kingdom

## Abstract

**Background:**

Given the increasing burden of chronic conditions, multimorbidity is now a priority for healthcare and public health systems worldwide. Appropriate methodological approaches for assessing the phenomenon have not yet been established, resulting in inconsistent and incomplete descriptions. We aimed to estimate and characterize the burden of multimorbidity in the adult population in France in terms of number and type of conditions, type of underlying mechanisms, and analysis of the joint effects for identifying combinations with the most deleterious interaction effects on health status.

**Methods and findings:**

We used a multistep approach to analyze cross-sectional and longitudinal data from 2 large nationwide representative surveys: 2010/2014 waves of the Health, Health Care, and Insurance Survey (ESPS 2010–2014) and Disability Healthcare Household Survey 2008 (HSM 2008), that collected similar data on 61 chronic or recurrent conditions. Adults aged ≥25 years in either ESPS 2010 (14,875) or HSM 2008 (23,348) were considered (participation rates were 65% and 62%, respectively). Longitudinal analyses included 7,438 participants of ESPS 2010 with follow-up for mortality (97%) of whom 3,798 were reinterviewed in 2014 (52%). Mortality, activity limitation, self-reported health, difficulties in activities/instrumental activities of daily living, and Medical Outcomes Study Short-Form 12-Item Health Survey were the health status measures. Multiple regression models were used to estimate the impact of chronic or recurrent conditions and multimorbid associations (dyads, triads, and tetrads) on health status. Etiological pathways explaining associations were investigated, and joint effects and interactions between conditions on health status measures were evaluated using both additive and multiplicative scales.

Forty-eight chronic or recurrent conditions had an independent impact on mortality, activity limitations, or perceived heath. Multimorbidity prevalence varied between 30% (1-year time frame) and 39% (lifetime frame), and more markedly according to sex (higher in women), age (with greatest increases in middle-aged), and socioeconomic status (higher in less educated and low-income individuals and manual workers). We identified various multimorbid combinations, mostly involving vasculometabolic and musculoskeletal conditions and mental disorders, which could be explained by direct causation, shared or associated risk factors, or less frequently, confounding or chance. Combinations with the highest health impacts included diseases with complications but also associations of conditions affecting systems involved in locomotion and sensorial functions (impact on activity limitations), and associations including mental disorders (impact on perceived health). The interaction effects of the associated conditions varied on a continuum from subadditive and additive (associations involving cardiometabolic conditions, low back pain, osteoporosis, injury sequelae, depression, and anxiety) to multiplicative and supermultiplicative (associations involving obesity, chronic obstructive pulmonary disease, migraine, and certain osteoarticular pathologies). Study limitations included self-reported information on chronic conditions and the insufficient power of some analyses.

**Conclusions:**

Multimorbidity assessments should move beyond simply counting conditions and take into account the variable impacts on health status, etiological pathways, and joint effects of associated conditions. In particular, the multimorbid combinations with substantial health impacts or shared risk factors deserve closer attention. Our findings also suggest that multimorbidity assessment and management may be beneficial already in midlife and probably earlier in disadvantaged groups.

## Author summary

**Why was this study done?:**

- The growing burden of chronic conditions has made multimorbidity a priority for healthcare and public health systems worldwide.
- Over the past 2 decades, multimorbidity has been increasingly investigated in various settings and populations, but the large variations in estimates can partly be attributed to the heterogeneity of settings, designs, and analytical methods.
- The impact and joint effects of the most frequently associated chronic or recurrent conditions on health have not yet been quantified, and the underlying mechanisms explaining these associations have been seldom explored.

**What did the researchers do and find?:**

- A comprehensive and multistep analysis of 2 large nationwide representative surveys allowed the estimation and characterization of the burden of multimorbidity in the general population in France.
- This study clarified several issues regarding the assessment and measurement of multimorbidity, notably in terms of the number and type of conditions to be considered, the type of underlying mechanisms to explain the associations, and the analysis of the joint effects of associated conditions to identify those with most deleterious interaction effects on health.

**What do these findings mean?:**

- Multimorbidity assessments should move beyond simply counting chronic conditions and include a wide spectrum of chronic conditions. They should especially consider the variable impacts and joint effects of associated conditions.
- Multimorbid combinations with large health impacts or multiplicative interactions should also be appropriately prioritized for monitoring and management. As multimorbidity does not only affect older people, its assessment and management should begin in midlife and probably earlier in disadvantaged groups.

## Introduction

The gains in life expectancy observed worldwide have increased the risk of living with chronic conditions [1]. Over the past 2 decades, multimorbidity, defined as the co-occurrence of at least 2 chronic or recurrent conditions [2], has been increasingly investigated and estimated in various populations, settings, and geographical locations [3–6]. The broad variations observed in the available estimates of the burden of multimorbidity can be attributed, at least partly, to heterogeneous study methodologies in terms of the number and type of conditions (e.g., diseases, risk factors, symptoms, specific conditions) as well as the thresholds used (e.g., two or three) [7,8]. In particular, the identification of conditions for inclusion in multimorbidity assessments has largely relied on the prevalence, or more commonly, the availability of conditions in existing datasets [8]. The type and meaning of multimorbid associations have subsequently received attention [9], although the etiological pathways have seldom been explored [10]. Significantly, the quantified impact of multimorbid associations on relevant health indicators such as mortality or limitations in daily activities has scarcely been estimated [11,12]. Furthermore, the metrics of the joint effects of the associated conditions (i.e., additive or multiplicative scale), which is a critical issue for measuring multimorbidity, has been largely ignored.

To address this gap, we aimed to estimate and characterize the burden of multimorbidity in the French general population using a comprehensive and multistep analytical approach in order to (1) identify the relevant chronic or recurrent conditions for inclusion in multimorbidity assessments based on their impact on health status; (2) determine the frequency of the co-occurrence of these conditions and their underlying etiological pathways; and (3) evaluate the impact of the number of conditions and their associations as well as the joint effects of associated conditions on health. This study further aimed to contribute to our knowledge of multimorbidity in France where general population studies are rare [6].

## Materials and methods

### Survey designs and study populations

We used data from 2 large nationwide representative surveys conducted in France between 2008 and 2014, which used similar interview and sampling methods as well as similar lists of chronic conditions and health indicators: the Health, Health Care, and Insurance Survey from 2010 and 2014 (Enquête Santé et Protection Sociale, ESPS) and the Disability Healthcare Household Survey from 2008 (Enquête Handicap–Santé Ménages, HSM). Despite several differences, notably in terms of the time frame used to investigate morbidities that prevented cross-validation or replication in the strictest sense, there was sufficient commonality between the studies to look for convergent evidence based on their parallel analyses.

ESPS is a longitudinal health survey representative of individuals living in households in France (95% of the total population). It collects information about their health status through telephone and face-to-face interviews conducted by specially trained interviewers as well as self-administered questionnaires [13]. In 2010, the participation rate was 65%, resulting in 14,875 participants aged ≥25 years. Half of participants (*N =* 7,727, one per household) were scheduled to be followed-up to be reinterviewed regarding health status measures in 2014. Linkage with vital statistics, for these participants only, also allowed for the assessment of mortality up to and including 2014.

HSM is a purely cross-sectional two-stage survey conducted in 2008 with a focus on health, disability, and dependency. The participation rates for the first stage to screen individuals with disabilities and the second stage were 80% and 77%, respectively, leading to 23,348 participants aged ≥25 years residing in France being evaluated in face-to-face interviews and self-administered questionnaires in the second stage [14].

Both HSM 2008 and ESPS 2010–2014 received institutional review board approval, and participants provided written informed consent.

### Chronic and recurrent conditions

Both surveys used similar interviews and questionnaires, although there were minor variations in the lists and labels of self-reported conditions. A total of 61 chronic or recurrent conditions were recorded, which is at the top end of the range (25 to 75) recommended by Holzer and colleagues for multimorbidy assessement [15]. This list included conditions typically found in previous multimorbidity studies [15] (Table 1). The ESPS survey considered conditions occurring within the past 1-year period, whereas the HSM survey considered lifetime occurrence. Both time frames are relevant, as several conditions have lifelong implications (e.g., cancer, vascular diseases), while others have more temporary effects (e.g., depression, asthma) [5].

**Table 1:** Frequency and associations of conditions with activity limitations, perceived health, or mortality in the ESPS and HSM surveys. An X indicates that the condition was independently associated with the health status measure. All estimates are weighted to represent the French general population estimates. The 48 conditions selected for further analysis are highlighted in bold.

	1-year time frame (ESPS Survey)										Lifetime frame (HSM Survey)									
	Descriptive statistics			Health status measures independently associated with the condition, cross-sectional analyses				Health status measures independently associated with the condition, longitudinal analyses			Descriptive statistics			Health status measures independently associated with the condition, cross-sectional analyses						
	Frequency of the condition (%)	Male-to-female ratio	Median age of participants reporting the condition	GALI limited, severely	GALI limited, not severely	Bad or very bad health	Fair health	Death between 2010 and 2014	New limitation**	New health deterioration***	Frequency of the condition (%)	Male to female ratio	Median age of participants reporting the condition	GALI limited, severely	GALI limited, not severely	Bad or very bad health	Fair health	Limitation in ≥3 ADLs or ≥2 IADLs	Limitation in <3 ADLs and <2 IADLs	SF-12 total score
**HIV infection**	0.02	0.58	60								0.03	0.38	51			X	X			
**Colorectal cancer**	0.38	0.94	72	X	X	X	X	X			0.32	0.68	68	X		X	X			X
**Oropharyngeal and laryngeal cancer**	0.11	1.65	69	X		X	X				0.28	1.72	61	X	X	X	X	X	X	X
**Lung cancer**	0.12	3.03	65	X	X	X		X			0.21	3.36	60	X	X	X	X	X	X	X
Skin cancer	0.23	0.52	64								0.32	1.08	66							
**Breast cancer**	1.05	–	62	X	X	X	X	X			1.30	–	62	X	X	X	X		X	X
Uterus cancer (endometrial and cervical)	0.28	–	62								0.52	–	58							
**Prostate cancer**	0.76	–	71								0.67	–	72			X				
**Urinary tract cancer (kidney, bladder)**	0.18	1.85	65			X	X				0.25	2.91	62	X	X	X	X			X
**Other cancer**	0.50	1.26	63	X	X	X	X	X			0.78	0.61	59	X	X	X	X	X	X	
**Malignant hemopathies**	0.39	0.84	63	X	X	X				X	0.26	0.84	57	X	X	X	X	X		X
Nutritional anemias	0.16	0.20	42	X							0.03	0.40	55							
Bleeding disorders	0.04	0.47	39								0.12	1.49	44							
**Thyroid disorders**	4.50	0.12	60				X				5.32	0.14	57				X			
**Diabetes**	4.43	1.06	67	X	X	X	X	X			6.15	1.11	64			X	X	X	X	X
**Obesity (morbid, BMI >35)†**	0.54	0.30	57	X	X	X)	X				0.84	0.32	53	X	X	X	X	X	X	X
**Obesity (nonmorbid, BMI 30–35)†**	8.85	0.86	56	X	X	X	X		X	X	12.42	0.86	55	X	X	X	X	X	X	X
Metabolic abnormalities (hyperlipidemia)	12.36	0.92	64								1.99	1.13	58							
**Substance abuse**	0.20	1.71	41					X			0.05	1.98	35	X	X	X	X		X	X
**Schizophrenia**	0.05	0.60	58	X							0.13	1.98	38	X	X	X	X	X	X	X
**Depression**	5.23	0.37	55	X	X	X	X				4.21	0.53	54	X	X	X	X	X	X	X
**Anxiety**	8.91	0.41	55	X	X	X	X				5.49	0.51	55	X	X	X	X	X	X	X
**Parkinson’s disease**	0.26	1.58	71	X		X			X		0.40	1.46	75	X	X	X	X	X	X	X
**Alzheimer’s disease and other dementias**	0.21	0.89	78	X	X	X	X	X			0.52	0.57	81	X	X	X	X	X)	X	X
**Multiple sclerosis**	0.10	0.22	48	X	X	X	X				0.25	0.35	51	X	X	X	X	X	X	X
**Epilepsy**	0.66	1.70	56	X		X	X				0.92	1.17	44	X	X	X	X	X	X	
**Migraine**	6.43	0.32	46		X	X	X				9.56	0.44	47	X	X	X	X	X	X	X
**Cataract**	3.20	0.58	76								4.61	0.53	77					X	X	
**Iris, choroidal, and retinal diseases**	0.72	0.68	67	X	X	X					0.80	0.65	64	X	X			X	X	
**Glaucoma**	1.69	0.51	69							X	1.42	0.70	69	X	X			X		X
**Ear ailments**	7.83	0.96	66	X	X	X	X				5.18	1.14	58			X	X			
**Hypertension**	14.66	0.85	67			X	X				14.64	0.75	64		X	X	X			
**Ischemic heart disease**	1.35	1.07	72		X	X	X				2.83	1.62	72	X	X	X	X	X	X	X
**Myocardial infarction**	0.99	3.29	71	X	X	X					1.78	3.36	70	X	X	X	X			X
**Cardiac rhythm disorders**	4.06	0.78	70	X	X	X	X				3.09	0.78	65	X		X	X			X
**Heart failure**	0.38	2.47	75								2.42	1.02	74	X	X	X	X	X	X	X
**Stroke**	1.19	1.52	70	X	X	X	X				1.61	1.09	73	X	X	X	X	X	X	X
**Peripheral arterial disease**	1.21	1.45	70	X		X					1.14	1.93	69	X	X	X	X			X
Lower extremity varices	8.27	0.33	61								9.14	0.27	59							
Hemorrhoids	5.71	0.74	53								5.62	0.75	53							
Allergic rhinitis	6.46	0.58	48								8.18	0.61	45							
**Chronic obstructive pulmonary disease**	2.05	0.84	64	X		X	X		X	X	4.68	1.05	62	X	X	X	X	X	X	X
**Asthma**	3.34	0.57	48	X	X	X	X				6.35	0.81	46	X	X	X	X		X	X
**Peptic ulcer**	1.67	1.23	59	X		X	X				3.51	1.07	56	X		X	X	X	X	X
**Inflammatory bowel diseases**	2.58	0.50	50								0.37	1.24	48	X	X	X				
Food allergies	0.03	0.00	53								1.39	0.39	48							
**Chronic liver diseases**	0.61	0.90	55	X	X	X	X	X			0.64	0.76	59	X	X	X	X			X
Eczema	3.14	0.60	44								6.01	0.52	45							
Psoriasis	2.84	0.80	52								3.28	0.97	49							
**Rheumatoid arthritis**	0.34	0.60	62	X	X	X	X			X	1.74	0.49	65	X	X	X	X	X	X	X
**Other (nonrheumatoid) inflammatory arthritis**	0.79	1.18	64	X	X	X	X				3.45	0.63	58	X	X	X	X	X	X	X
**Osteoarthritis of the hip**	4.04	0.63	68	X	X	X	X				5.02	0.61	68	X	X	X	X	X	X)	X
**Osteoarthritis of the knee**	8.00	0.66	66	X	X	X	X		X	X	8.16	0.60	66	X	X	X	X	X		X
**Osteoarthritis of other peripheral joints**	6.98	0.45	63	X	X	X	X		X	X	9.89	0.49	63	X	X	X	X	X	X	X
**Low back pain**	12.98	0.78	56	X	X	X	X		X	X	17.66	0.88	53	X	X	X	X		X	X
**Osteoporosis**	2.94	0.08	68	X		X	X				2.79	0.10	69	X		X	X			X
**Kidney failure**	0.13	0.66	63	X	X	X	X				0.22	2.50	68	X	X	X		X	X	
Repeated urinary infections	2.67	0.15	54								3.65	0.11	51							
**Urinary incontinence**	0.30	0.31	63	X	X						2.87	0.31	72	X	X	X	X	X		X
Benign prostatic hypertrophy	2.04	–	72								1.57	–	73							
**Injury sequelae**	1.27	1.16	55	X	X	X	X		X	X	6.09	1.56	52	X	X	X	X	X	X	X

ADL, limitation in activity of daily living; BMI, body mass index; ESPS, Enquête Santé et Protection Sociale; GALI, Global Activity Limitation Indicator; HIV, human immunodeficiency virus; HSM, Enquête Handicap–Santé Ménages; IADL, limitation in instrumental activity of daily living; SF-12, Medical Outcomes Study Short-Form 12-Item Health Survey; SRH, Self-Reported Health indicator.

*Polytomous logistic regression using “no limitation” or “good or very good heath” or “no limitation in ADL/IADL” as the reference category. All models include age and sex.

**Limitation, severe or not in 2014 in participants who were not limited in 2010.

***Health graded less than good in 2014 in participants with good/very good health in 2010.

†Obesity was categorized according to the standard BMI criteria (obese: BMI 30–35; morbidly obese: BMI >35) and analyzed as a 3-category variable.

### Health status measures

**Fig 1:**
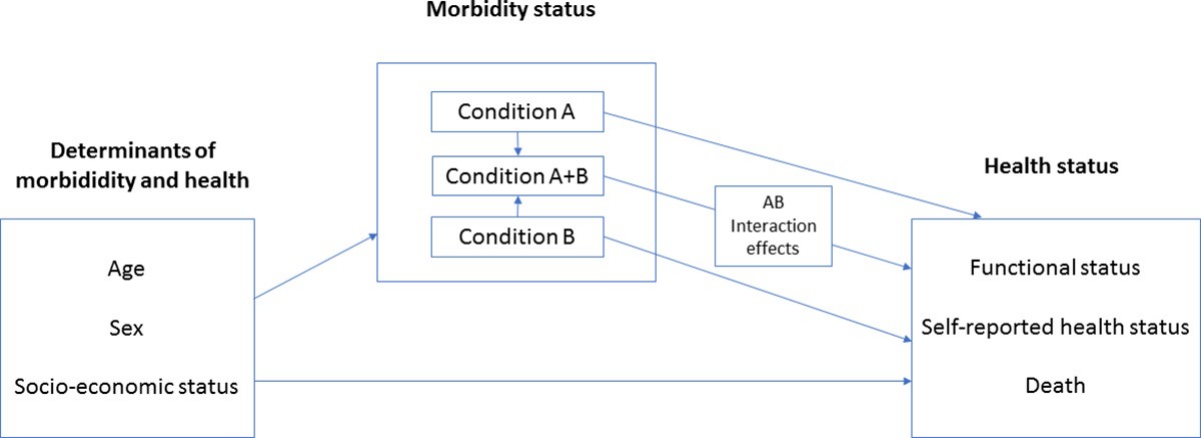
Schematic diagram of the conceptual model assumed in this study.

In addition to the mortality recorded in the ESPS survey, several measurements relating to functioning and perceived health were taken into account using valid and reliable instruments. Both surveys included the European Union Global Activity Limitation Indicator (GALI; extent to which the participant is limited because of a health problem in their daily activities: severely limited/limited but not severely/not limited at all) and Self-Reported Health (SRH; how the participant evaluates his/her general health: very good/good/fair/bad/very bad) [16]. Difficulties in activities of daily living (ADL, *N =* 7) [17], instrumental activities of daily living (IADL, *N* = 12) [18], and the Medical Outcomes Study Short-Form 12-Item Health Survey (SF-12) questionnaire were only included in the HSM survey [19]. Change scores were calculated for functioning and perceived health measures in ESPS participants when repeated measurements were available (GALI, SRH). Fig 1 presents the conceptual model assumed in this study. In line with a large body of research in the field of disability and perceived health and quality of life [20–22], the pathological processes were considered to cause disability and nonoptimal health; age, sex, and socioeconomic status were considered to be the determinants of morbid conditions, disability, and nonoptimal health. Under this model, it is possible to evaluate and quantify the impacts of morbid conditions on health status.

### Statistical analysis

We implemented a sequential multistep analytical approach to (1) identify the relevant conditions for inclusion in the multimorbidity analysis; (2) determine the frequency and type of associations of conditions identified as relevant; and (3) estimate the impact of these multimorbid associations on health status.

#### Identifying the relevant conditions associated with impacts on health status

We aimed to select conditions that were consistently associated with independent impacts on health status across surveys and/or within surveys across indicators (functioning, perceived health, mortality) and across thresholds in the case of categorical indicators (see below). Multiple regression models were used to assess the independent effects on health status measures of each recorded condition adjusted for age and sex: binary logistic regression for mortality, new limitation in GALI, and health deterioration in SRH; polytomous logistic regression for categorical indicators in GALI (two thresholds: any and only severe limitation), SRH (two thresholds: “fair health” and “good health”), and ADL-IADL (two thresholds: any and limitation in ≥3 ADLs or ≥2 IADLs); and linear regression for SF-12 summary scores. As recommended for over a decade [23–25], lasso methods were used to select the conditions to be retained in the final models. Interactions with age and sex were tested for each condition included in the final models. We also compared the explanatory power of the raw count of included conditions relative to the inclusion of binary indicators for all conditions; we used restricted cubic splines to model nonlinear relationships between the count and the health status measures using 5 knots: 2, 3, 4, 6, and 9, respectively, located at the 35 or 50th, 60 or 70th, 88th, 95th, and 99th percentiles of the number of morbidities according to the study.

#### Determining the frequency and type of associated conditions

**Fig 2:**
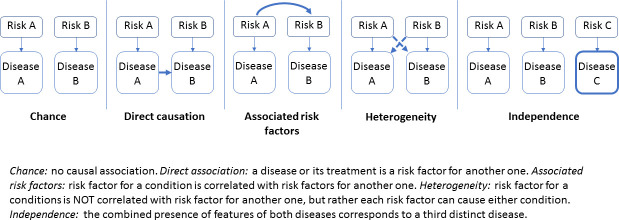
Typology of etiological associations between comorbid conditions.

We described the frequency of dyads, triads, and tetrads of the associated conditions retained in the previous step and investigated the etiological pathways that most plausibly explain the most frequent associations (≥0.25%) using the typology by Valderas and colleagues [10] who retained 5 pathways: direct causation, shared risk factors, associated risk factors, confounding by another condition, and only chance (Fig 2). This categorization was practically implemented in a standardized way, through the following steps:

- The age- and sex-adjusted odds ratio (OR) for the dyad and the fully adjusted OR, including all conditions independently associated with both components of the dyad, were computed using binary logistic models (with the first component of the dyad as the dependent variable). If the 95% confidence interval (CI) of the age- and sex-adjusted OR included 1, we considered that the dyad could be “explained by chance.” If the 95% CI of the age- and sex-adjusted OR did not include 1, while the 95% CI of the fully adjusted OR included 1, we considered that the dyad could be explained by confounding by another condition. These analyses were conducted in both ESPS and HSM surveys.
- When the 95% CI of the fully adjusted OR for the dyad did not include 1 (independent association) in both surveys, a thorough literature search was performed by one of us (JC) on MEDLINE and Google Scholar search engines using the following terms: “association,” “cause,” “determinant,” “complication,” “risk factor,” or “epidemiology” along with each component of the dyad. Systematic reviews as well as primary studies were considered. If a reasonable consensus was found in the literature regarding a causal link between the conditions, we considered that the dyad could be explained by a causal relationship. We subsequently specified whether this relation was bidirectional or due to a complication. Otherwise, if there was a reasonable consensus regarding the shared risk factors, we considered that the dyad could be explained by “shared risk factors” and thus mentioned them. Otherwise, if there was a reasonable consensus regarding the association of risk factors, we considered that the dyad could be explained by “associated risk factors” and thus mentioned them. Several exceptions were made to this categorization to indicate “strongly related pathological processes” such as hip and knee osteoarthritis, ischemic heart disease and peripheral arterial disease (atherosclerosis), or anxiety and depression.

#### Estimating the impact and joint effects of multimorbid associations

**Fig 3:**
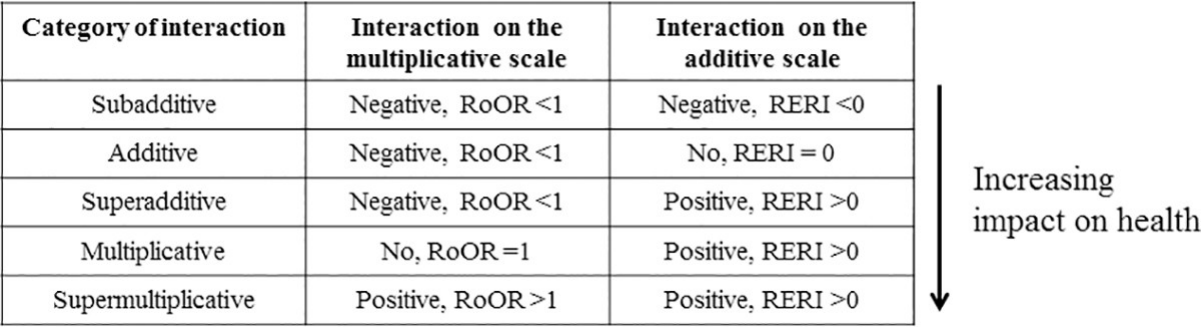
Interaction continuum and 5 categories of interaction on additive and multiplicative scales considered in this study. RoOR, ratio of odds ratios; RERI, relative excess risk due to interaction.

We described the health impacts of dyads, triads, and tetrads of associated conditions on activities, perceived health, and mortality. Following VanderWeele [26], the joint effects of multimorbid associations were conceptualized as the sum of the effects of each condition and their interaction, with the latter considered on a continuum ranging from subadditive (negative additive interaction) to supermultiplicative (positive multiplicative interaction). The placement on the continuum depended on the relative magnitude of the probability of negative health status in the group in terms of the associated conditions: Associations at the (super)multiplicative end are associated with worse health status than those at the (sub)additive end (Fig 3).

Joint effects and interactions of associated conditions on functioning (severely limited in GALI; limited in ≥3 ADLs or ≥2 IADLs; new limitation) and perceived health (bad or very bad; health deterioration) were thus evaluated on both additive and multiplicative scales by estimating the relative excess risk due to interaction (RERI) [27] and the ratio of odds ratios (RoOR) in the logistic models, including the associations of conditions, age, sex, and all the conditions independently associated with the predicted health indicator as identified in the first step. Due to a limited number of events, it was not possible to study the joints effects on mortality. To ensure that the RoOR and RERI estimates (based on the ORs) were valid, joint-effect analyses were only conducted for health indicators with a prevalence ≤10% (the ORs approximate the relative risks in this context). CIs of RERI were computed according to Zou [28].

All the analyses (descriptive and analytic) were performed separately for the 2 surveys using SAS, version 9.2 software (SAS Institute, Cary, NC, USA) and R software, version 3.6.1 (lasso methods). Appropriate weights were used to provide valid estimates for the French population, while taking into account the unequal probabilities of selection resulting from sample design, nonresponse, and noncoverage in the 2 surveys [13,14]. Due to the data collection methodology used (mostly personal interviews in the 2 surveys), there were very few missing data (<0.005) either for conditions, standard sociodemographic variables, or health status measures. Due to the complex sampling designs, however, classical standard goodness of fit and predictive ability statistics including the Hosmer–Lemeshow statistic and c-index were not available. The Akaike information criterion (AIC) was used for model comparison.

The main analyses were planned before being conducted. Further analyses were only performed to respond to the reviewers’ comments, notably regarding the characteristics of ESPS participants in the longitudinal analyses and the risk of multimorbidity associated with sociodemographic variables, with no influence on the main findings. No data-driven changes were made to the analyses.

This study is reported in line with the Strengthening the Reporting of Observational Studies in Epidemiology (STROBE) guidelines (S1 STROBE Checklist).

## Results

All 14,875 ESPS and 23,348 HSM participants aged ≥25 were included in the cross-sectional analyses. Nearly half of the ESPS sample (*N =* 7,438, 97% of scheduled participants) was followed up for mortality until 2014, when 3,798 (52%) agreed to participate again in the survey for longitudinal analyses. A description of the 4 analyzed samples is given in S1 Table. There were no important differences between them.

### Chronic or recurrent conditions with a negative impact on health status

Overall, 48 out of 61 recorded chronic or recurrent conditions consistently affected activity limitations, perceived health, or mortality (Table 1 and S2 Table). The remaining conditions were not associated with any of the health status measures, with the exception of nutritional anemias, which had an inconsistent impact on GALI. There was no interaction of sex with any assessed risk. An interaction of age was observed for low back pain, depression, and injury sequelae: Younger participants with these conditions had a higher risk of limitations or bad health than older ones. S3 Table details the impact of the studied chronic conditions on functioning and perceived health within the 2 reference time frames (1-year versus lifetime). Although generally similar, the proportion of participants reporting more severe impacts tended to be higher within the lifetime frame (a difference not explained by the age of the affected participants).

The raw count of these 48 conditions was associated with all health status measures (except mortality), although it was less informative than the separate inclusion of all the conditions (AIC 5% to 10% higher than those obtained with the final models). Moreover, the changes in slope (negative) observed for all indicators except for one at 3 and 6 conditions in spline regressions suggest that the health impacts rapidly stabilize, probably due to ceiling effects, when using this count (see below).

### Prevalence of, and factors associated with multimorbidity

Overall, multimorbidity was observed in 30.4% of participants (95% CI: 29.6% to 31.3%) using the 1-year time frame of the ESPS survey and 39.0% (95% CI: 38.0% to 40.0%) using the lifetime frame of the HSM survey (Table 2). Regardless of the time frame, the prevalence of multimorbidity increased almost linearly with age and was higher in women than in men: Using the 1-year time frame, it ranged from 7% among 25- to 34-year-old men to 67% among women over 85 years. The greatest increases in prevalence were observed between the age groups of 45 to 54 and 55 to 64 years, and 55 to 64 and 65 to 74 years. Prevalence was also higher in less educated and low-income participants and manual workers, particularly in women and middle-aged participants. Multiple logistic regression analyses confirmed the independent and “dose-effect” associations of age, sex, education level, occupation, and household income with multimorbidity (S4 Table).

**Table 2:** Weighted frequency of multimorbidity, defined as having at least 2 of the 48 selected conditions, analyzed according to age, sex, and 3 socioeconomic status indicators (education level, occupation, and household income). Conditions present during the last 12 months (ESPS Survey) and lifetime presence (HSM Survey).

		1-year time frame (ESPS Survey)/Lifetime frame (HSM Survey)						
		25–34 y	35–44 y	45–54 y	55–64 y	65–74 y	75–84 y	≥85 y
**Men**	All	7.1/12.2	10.9/20.0	18.2/31.9	36.7/45.5	51.0/63.1	66.2/68.7	62.4/78.3
	Less than secondary education	5.9/17.0	11.7/28.8	20.6/37.6	37.8/51.1	53.5/66.1	63.3/72.1	62.7/75.6
	Secondary education	7.7/13.3	12.3/20.8	19.1/33.2	37.8/44.6	48.0/62.0	74.6/66.5	53.1/88.6
	Tertiary education	6.7/9.6	8.6/13.2	13.1/22.8	32.5/37.7	51.0/55.6	67.1/NC	85.0/NC
	Occupation, other or manual worker	7.5/15.2	10.3/23.7	18.8/35.4	39.2/50.7	51.5/68.2	64.0/69.5	56.9/76.0
	Occupation, middle manager or teacher	4.9/9.5	15.1/21.5	19.3/30.5	35.2/45.0	49.5/61.9	75.7/70.7	83.2/87.5
	Occupation, manager or professional	9.1/9.3	5.8/9.9	14.8/25.8	33.2/36.9	52.7/53.9	65.6/62.7	65.7/75.6
	Household income, lower third	8.3/15.2	20.5/25.0	28.7/39.5	47.1/54.2	61.7/67.6	70.6/69.0	75.4/80.8
	Household income, middle third	7.4/12.4	12.4/20.9	21.8/32.3	51.6/44.8	62.7/65.1	79.3/72.6	76.5/70.8
	Household income, upper third	8.9/10.9	10.9/16.5	20.1/28.5	38.3/38.8	60.0/60.9	80.6/62.6	NC/88.3
**Women**	All	13.3/15.9	20.2/24.1	30.7/38.2	40.6/52.6	58.7/66.6	70.3/76.4	67.0/77.9
	Less than secondary education	15.9/25.0	21.4/32.6	33.7/50.2	43.8/60.6	57.5/72.4	68.8/76.3	67.1/81.8
	Secondary education	10.7/15.5	20.9/26.0	30.3/36.0	39.9/50.6	61.9/59.3	77.4/75.1	63.5/71.2
	Tertiary education	15.9/14.4	18.8/17.9	27.2/30.3	33.7/39.8	57.0/55.3	73.2/85.5	74.4/NC
	Occupation, other or manual worker	15.2/19.3	20.4/30.8	31.6/43.7	40.5/56.0	60.5/71.4	68.3/77.2	66.3/84.3
	Occupation, middle manager or teacher	13.6/15.3	21.5/22.2	31.3/36.9	43.4/54.1	59.3/64.9	71.8/75.7	72.3/71.1
	Occupation, manager or professional	8.0/12.2	15.7/10.5	23.1/26.0	32.2/38.1	53.3/54.2	81.1/NC	72.6/NC
	Household income, lower third	21.4/19.7	29.7/32.2	50.7/48.4	54.6/61.8	74.0/68.6	79.0/77.7	79.6/81.2
	Household income, middle third	15.6/13.6	28.2/23.9	38.6/34.6	46.9/51.4	71.7/65.9	80.9/80.2	80.3/78.2
	Household income, upper third	11.1/16.8	20.0/20.2	30.8/36.5	48.2/44.0	66.2/64.9	82.8/78.3	NC/78.6

ESPS, Enquête Santé et Protection Sociale; HSM, Enquête Handicap–Santé Ménages; NC, estimation not calculated due to limited sample size.

The vast majority of participants reporting multiple conditions had up to 4 conditions (74% and 78% in the ESPS and HSM surveys, respectively) (S5 Table). The presence of 6 or more concomitant conditions was uncommon before 75 years but was observed in one-quarter of women after 85 years.

Regardless of participant age and survey time frame, about three-quarters of conditions were more often observed in multimorbid combinations than alone, while 24 and 4 conditions had a median of 2 and 3 associated conditions, respectively (S6 Table).

### Associations of conditions and type of multimorbid combinations

**Fig 4:**
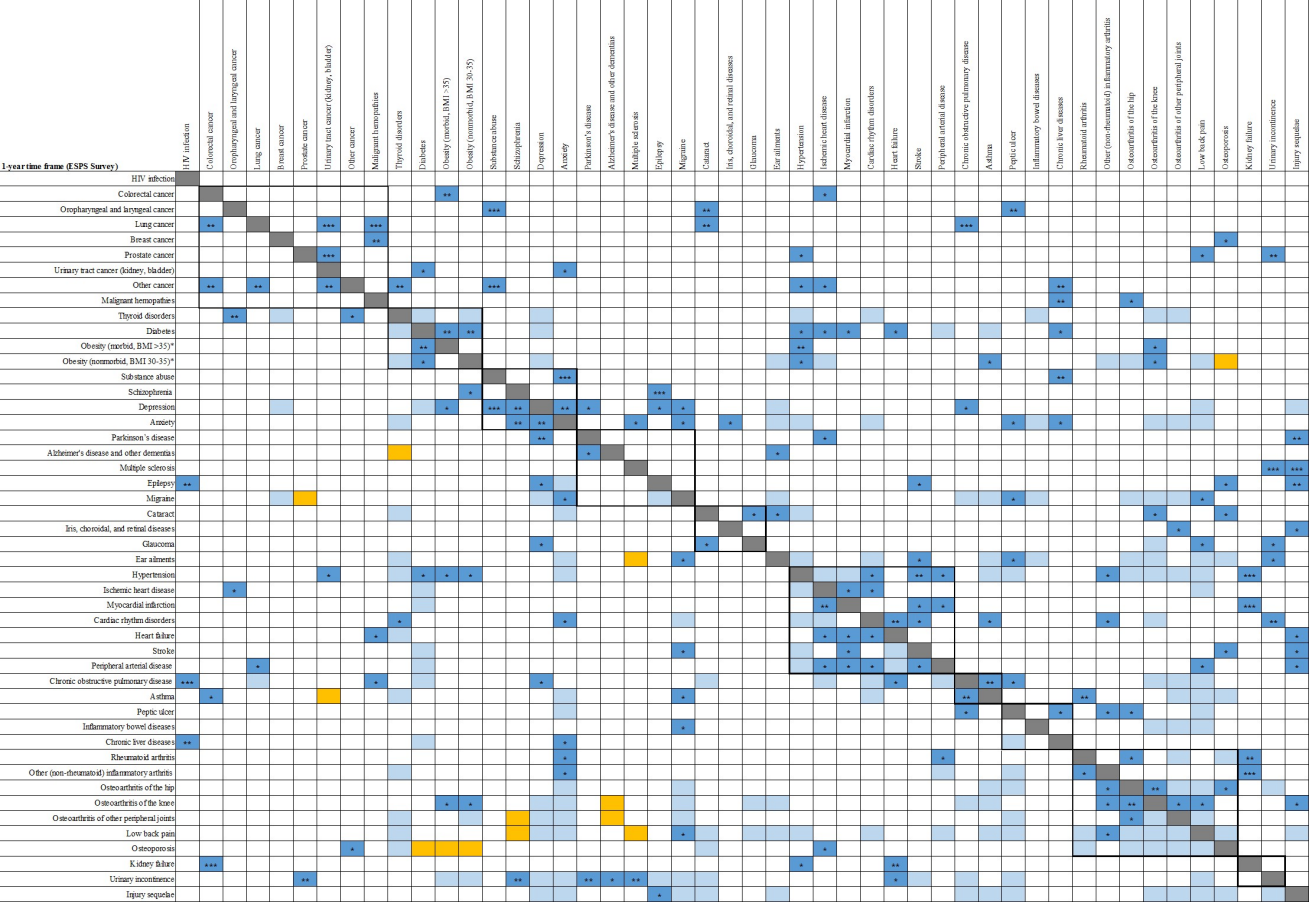
Independent associations between the 48 selected chronic conditions. Associations were identified in logistic models built with forward and backward stepwise procedures to identify the conditions independently associated with each condition while adjusting for age and sex. Upper right panel, above the diagonal: ESPS survey and 1-year time frame; lower left panel, below the diagonal: HSM survey and lifetime frame. Obesity is categorized according to the standard BMI criteria (obese: BMI 30–35; morbidly obese: BMI >35) and analyzed as a 3-category variable. A light blue box indicates an OR ≤2. A star sign (*) indicates an OR between 2 and 4; 2 stars (**) indicate an OR between 4 and 10; 3 stars (***) indicate an OR ≥10. An orange box indicates a negative association (OR <1). Frames indicate conditions within the same ICD-10 chapter. BMI, body mass index; ESPS, Enquête Santé et Protection Sociale; HIV, human immunodeficiency virus; HSM, Enquête Handicap–Santé Ménages; ICD-10, 10th revision of the International Statistical Classification of Diseases and Related Health Problems; OR, odds ratio.

The most frequent dyads (*N* = 223), triads and tetrads (*N* = 76) of chronic conditions along with the plausible etiological pathways explaining their associations are presented in Table 3 and S7 and S8 Tables. The most frequent combinations include hypertension, low back pain, obesity, osteoarthritis (knee, hip, or other peripheral joints), migraine, diabetes, anxiety, depression, and ear ailments. Fig 4 summarizes the two-by-two independent associations (dyads) between the 48 selected chronic conditions by visualizing 3 main aggregates of associations that are consistent across time frames: (i) vasculometabolic, including diseases of the circulatory system, diabetes, and obesity; (ii) diseases of the musculoskeletal system; and (iii) mental disorders. However, more scattered associations also exist, namely, anxiety and depression with various somatic conditions, especially musculoskeletal disorders, or the latter with vasculometabolic conditions.

**Table 3:** Weighted frequency of dyads of conditions, strength of associations, and plausible etiological pathways explaining the associations. All dyads with a frequency of ≥1% in both survey samples are considered. Dyads are presented in decreasing order of frequency (mean frequency based on the 2 surveys).

	1-year time frame (ESPS Survey)			Lifetime frame (HSM Survey)			
Dyad	Frequency	Age- and sex-adjusted OR	Fully adjusted* OR	Frequency	Age- and sex-adjusted OR	Fully adjusted* OR	Plausible etiological pathway(s) explaining the association
Hypertension-Low back pain	3.63	2.23 (1.94–2.58)	1.60 (1.36–1.88)	3.73	1.53 (1.36–1.72)	1.44 (1.27–1.63)	Shared determinants (obesity, smoking, mental)
Obesity, nonmorbid-Hypertension	3.40	4.22 (3.61–4.93)	2.98 (2.50–3.56)	3.50	2.49 (2.18–2.84)	2.24 (1.95–2.58)	Causal (determinant)
Hypertension-Osteoarthritis of the knee	3.56	2.65 (2.25–3.12)	1.61 (1.33–1.94)	2.41	1.38 (1.20–1.59)	1.09 (0.95–1.27)	Shared determinants (obesity, mental)
Osteoarthritis of the knee-Low back pain	3.03	4.03 (3.44–4.72)	2.63 (2.21–3.14)	2.88	2.42 (2.12–2.76)	1.67 (1.44–1.93)	Shared determinants (obesity, mental)
Migraine-Low back pain	2.08	4.03 (3.40–4.76)	2.45 (2.02–2.99)	3.37	3.11 (2.70–3.57)	2.41 (2.07–2.81)	Shared determinants (obesity, mental)
Osteoarthritis of other peripheral joints-Low back pain	2.30	2.99 (2.52–3.55)	2.59 (2.16–3.11)	3.15	2.10 (1.84–2.40)	1.52 (1.31–1.76)	Shared determinants (obesity, mental)
Hypertension-Osteoarthritis of other peripheral joints	2.74	2.32 (1.94–2.78)	2.02 (1.67–2.44)	2.56	1.24 (1.08–1.42)	1.13 (0.98–1.30)	Shared determinants (obesity, mental)
Anxiety-Low back pain	3.04	3.93 (3.39–4.56)	2.52 (2.12–3.00)	2.17	3.14 (2.69–3.67)	2.25 (1.91–2.65)	Causal (bidirectional)
Diabetes-Hypertension	2.67	5.53 (4.46–6.85)	3.99 (3.19–4.98)	2.42	2.75 (2.35–3.21)	2.13 (1.82–2.51)	Shared determinants (obesity)
Osteoarthritis of the knee-Osteoarthritis of other peripheral joints	2.11	3.57 (2.94–4.33)	2.08 (1.67–2.60)	2.50	2.83 (2.43–3.29)	2.02 (1.72–2.38)	Strongly related pathological processes
Osteoarthritis of the hip-Osteoarthritis of the knee	2.31	11.23 (8.99–14.02)	6.48 (5.10–8.25)	2.10	5.85 (4.94–6.92)	4.27 (3.60–5.06)	Strongly related pathological processes
Obesity, nonmorbid-Low back pain	2.04	1.98 (1.70–2.32)	1.33 (1.11–1.60)	2.35	1.04 (0.91–1.18)	-	Causal (determinant)
Ear ailments-Hypertension	3.02	2.08 (1.76–2.47)	1.36 (1.12–1.65)	1.16	1.30 (1.07–1.57)	1.19 (0.98–1.44)	Shared determinants (mental)
Depression-Anxiety	2.69	12.87 (10.65–15.56)	9.20 (7.45–11.37)	1.45	11.07 (9.13–13.42)	8.16 (6.61–10.06)	Strongly related pathological processes
Obesity, nonmorbid-Osteoarthritis of the knee	1.97	3.49 (2.91–4.17)	2.33 (1.89–2.87)	2.09	2.48 (2.15–2.86)	2.43 (2.10–2.81)	Causal (bidirectional)
Ear ailments-Low back pain	2.50	2.93 (2.49–3.45)	2.00 (1.68–2.40)	1.49	1.77 (1.49–2.11)	1.44 (1.19–1.74)	Shared determinants (mental)
Osteoarthritis of the hip-Low back pain	1.81	4.62 (3.78–5.66)	2.24 (1.76–2.86)	2.09	3.03 (2.57–3.56)	2.16 (1.81–2.57)	Shared determinants (obesity, mental)
Anxiety-Hypertension	2.51	2.17 (1.82–2.60)	1.57 (1.29–1.92)	1.35	1.61 (1.37–1.91)	1.39 (1.17–1.64)	Causal (bidirectional)
Migraine-Anxiety	1.95	5.11 (4.26–6.13)	3.43 (2.81–4.18)	1.60	4.41 (3.71–5.24)	2.77 (2.28–3.38)	Causal (bidirectional)
Hypertension-Osteoarthritis of the hip	1.97	2.63 (2.12–3.26)	1.42 (1.11–1.83)	1.52	1.31 (1.11–1.55)	1.17 (0.99–1.39)	Shared determinants (obesity)
Cataract-Hypertension	1.84	2.19 (1.72–2.80)	1.88 (1.45–2.43)	1.64	1.11 (0.94–1.32)	-	Causal (determinant)
Diabetes-Obesity, nonmorbid	1.48	5.11 (4.14–6.30)	3.58 (2.85–4.49)	1.94	3.23 (2.76–3.76)	2.83 (2.40–3.33)	Causal (determinant)
Hypertension-Cardiac rhythm disorders	2.18	3.34 (2.64–4.23)	2.68 (2.08–3.45)	1.05	1.91 (1.53–2.39)	1.62 (1.28–2.03)	Causal (determinant)
Depression-Low back pain	1.68	3.12 (2.58–3.77)	1.43 (1.14–1.79)	1.50	2.62 (2.22–3.09)	1.64 (1.37–1.98)	Causal (bidirectional)
Anxiety-Osteoarthritis of other peripheral joints	1.75	3.07 (2.54–3.72)	1.98 (1.59–2.42)	1.33	2.60 (2.17–3.12)	1.89 (1.56–2.29)	Causal (bidirectional)
Anxiety-Osteoarthritis of the knee	1.92	3.11 (2.57–3.75)	1.71 (1.36–2.15)	1.08	2.44 (2.04–2.93)	1.50 (1.22–1.83)	Causal (bidirectional)
Osteoarthritis of the hip-Osteoarthritis of other peripheral joints	1.24	3.49 (2.73–4.46)	1.69 (1.26–2.26)	1.76	3.14 (2.61–3.79)	2.20 (1.81–2.68)	Strongly related pathological processes
Thyroid disorders-Hypertension	1.57	2.37 (1.89–2.97)	1.79 (1.39–2.31)	1.39	1.70 (1.40–2.05)	1.49 (1.22–1.81)	Causal (complication)
Obesity, nonmorbid-Osteoarthritis of other peripheral joints	1.21	1.94 (1.57–2.39)	1.23 (0.96–1.57)	1.72	1.36 (1.18–1.58)	1.19 (1.03–1.39)	Causal (determinant)
Thyroid disorders-Low back pain	1.13	1.92 (1.55–2.39)	1.32 (1.03–1.70)	1.57	1.91 (1.60–2.27)	1.53 (1.07–1.85)	Causal (complication)
Migraine-Hypertension	1.10	1.70 (1.35–2.14)	1.11 (0.86–1.45)	1.52	1.28 (1.09–1.50)	1.07 (0.90–1.28)	Confounding
Cataract-Low back pain	1.09	2.30 (1.81–2.93)	1.25 (0.95–1.64)	1.42	1.58 (1.32–1.88)	1.37 (1.14–1.64)	Shared determinants (obesity, smoking)
Diabetes-Low back pain	1.04	1.58 (1.26–1.97)	1.12 (0.89–1.41)	1.35	1.10 (0.93–1.29)	-	Confounding
Cataract-Osteoarthritis of the knee	1.22	3.01 (2.34–3.88)	2.11 (1.60–2.78)	1.15	1.31 (1.09–1.59)	1.14 (0.94–1.39)	Shared determinants (obesity)
Depression-Migraine	1.19	4.72 (3.79–5.88)	2.10 (1.63–2.72)	1.16	3.86 (3.23–4.63)	1.95 (1.59–2.40)	Causal (bidirectional)
Cardiac rhythm disorders-Low back pain	1.25	2.29 (1.84–2.86)	1.49 (1.17–1.90)	1.03	2.04 (1.67–2.49)	1.48 (1.20–1.83)	Associated determinants (obesity, vascular)
Migraine-Osteoarthritis of the knee	1.01	3.07 (2.44–3.87)	1.61 (1.23–2.11)	1.25	2.17 (1.84–2.56)	1.59 (1.32–1.90)	Shared determinants (obesity, mental)
Diabetes-Osteoarthritis of the knee	1.12	2.16 (1.72–2.73)	1.23 (0.95–1.58)	1.08	1.57 (1.31–1.87)	1.20 (1.00–1.45)	Confounding

COPD, chronic obstructive pulmonary disease; ESPS, Enquête Santé et Protection Sociale; HSM, Enquête Handicap–Santé Ménages; NT, not tested due to a limited number of participants with the condition; OR, odds ratio.

*Full models include age-sex and all conditions associated independently with both components of the dyad (see Fig 1).

Overall, 44% of the most frequent two-by-two associations were found plausibly explicable by direct causation, either unidirectional as in the case of a complication (e.g., myocardial infarction and heart failure or hypertension and stroke) or bidirectional (e.g., osteoarthritis and obesity or depression and migraine). Furthermore, 30% can be plausibly explained by shared risk factors (e.g., chronic obstructive pulmonary disease (COPD) and ischemic heart disease), or more rarely, by associated risk factors (e.g., cardiac rhythm disorders and low back pain). Several associations (3%) may be explained by strongly related or similar pathological processes (e.g., ischemic heart disease and myocardial infarction or hip and knee osteoarthritis). Finally, for the remaining 23% of the associations, the co-occurrence of 2 conditions can, after adjustment, be attributed to chance (e.g., hypertension and osteoporosis) or confounding factors (e.g., heart failure and low back pain).

### Impact and joint effects of associations between conditions (dyads and triads)

The impact on health status varied greatly across the associated conditions (see S9 and S10 Tables for dyads and triads, respectively). With the exception of cardiovascular diseases complicated by heart failure and inflammatory arthritis by osteoarthritis, the combinations with the greatest impact on activities and general health frequently included conditions affecting different bodily systems, notably circulatory or respiratory, musculoskeletal, and nervous. Obesity, diabetes, eye and ear ailments, and mental disorders (especially depression) also contributed to the associations with the most impact. These impacts were consistent across time frames, although they were slightly greater in the lifetime frame.

**Fig 5:**
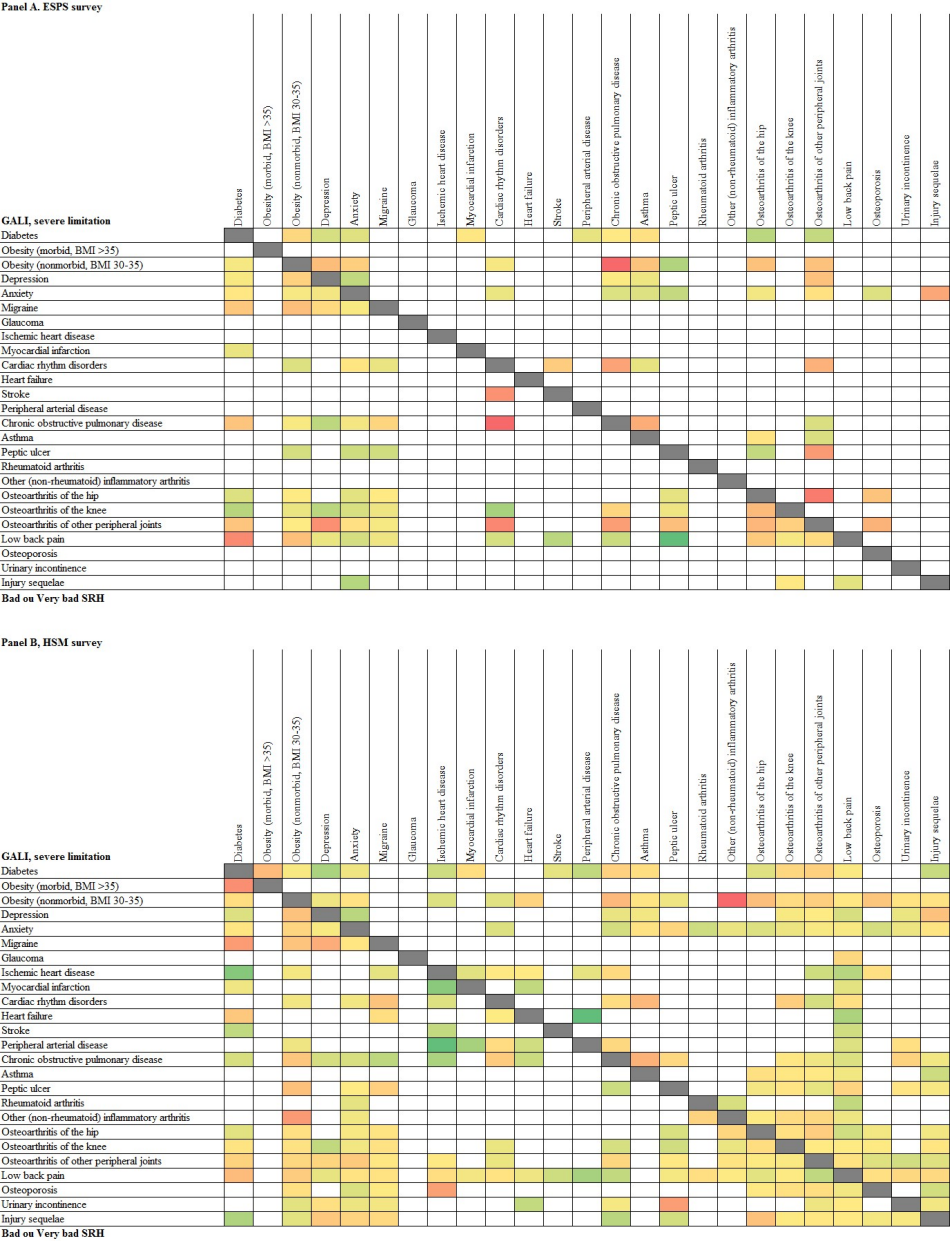
Interactions between the associated conditions in dyads on activity limitations (severely limited in GALI, upper right panel above the diagonal) and perceived health (bad or very bad SRH, lower left panel below the diagonal) in ESPS (Panel A) and HSM surveys (Panel B). Interactions are evaluated by the RERI (see S8 Table for values) and represented using a color scale from green (low values: subadditive or additive) to red (high values: multiplicative or supermultiplicative). Only dyads with a frequency of ≥0.25% in at least 1 survey sample are considered. BMI, body mass index; ESPS, Enquête Santé et Protection Sociale; GALI: Global Activity Limitation Indicator; HSM, Enquête Handicap–Santé Ménages; RERI, relative excess risk due to interaction; SRH: Self-Reported Health indicator.

Interactions between the associated conditions on health status ranged from subadditive to supermultiplicative (Fig 5 and S11 Table). Dyads with multiplicative or supermultiplicative interactions included obesity, chronic obstructive pulmonary disease, migraine accompanying other conditions, inflammatory arthritis and several forms of osteoarthritis, cardiac rhythm disorders, and stroke; dyads with additive interactions included cardiometabolic conditions, musculoskeletal conditions, and injury sequelae with other conditions. Depression and anxiety had a subadditive interaction: When associated with another condition, they had an additive interaction. The patterns of effects were similar across time frames, although the interactions shifted to the additive side for the lifetime frame compared to 1-year time frame (median RERI shift of 0.68 and 0.31 for activity limitations and perceived health, respectively). The same pattern of effects was observed within triads (S12 Table) where the joint effect of the third condition with the other two was similar to when the condition was separately associated with each of the other two.

## Discussion

The use of a comprehensive and multistep analytical approach to analyze 2 large representative general population samples with cross-sectional and longitudinal data on numerous chronic conditions and a broad range of health indicators allowed us to estimate and characterize the burden of multimorbidity in France. This study also makes novel contributions to 3 crucial aspects of multimorbidity assessments and measurements: (1) proposing a comprehensive method for estimating and characterizing the burden based on the health impacts; (2) determining the underlying mechanisms to explain the observed associations; and (3) identifying multimorbid combinations with a high impact and the most deleterious interaction effects on health.

### Burden of multimorbidity in France

Our study is the first to characterize the burden of multimorbidity in France. Estimates of the prevalence of specific conditions included in this study are similar to those previously reported in this country [29], while estimates of the prevalence of multimorbidity and specific multimorbid combinations as well as the observed higher prevalence among women are in line with other studies conducted in Europe [5,8] and worldwide [7]. Our study strongly confirms that multimorbidity is not just a problem of old age [30–33]: In middle age (35 to 54 years), it affects 1 in 6 men and 1 in 4 women. This study also found marked socioeconomic gradients, with participants, especially women, with lower education and socioeconomic status exhibiting higher multimorbidity rates earlier in the life, as already observed [5,34].

### Estimating and characterizing the burden of multimorbidity

This study showed that a large number of chronic conditions (almost 50) significantly and independently impact mortality, activity limitations, or perceived heath in adults. With the exception of the Global Burden of Diseases (GBD) program, which included over 200 noncommunicable diseases in its 2017 updated version [1], most public health agencies worldwide monitor much fewer chronic conditions. For example, the US Centers for Disease Control monitors 10 conditions in the Behavioral Risk Factor Surveillance System [35], while Public Health England generates prevalence estimates for 12 health problems according to “health profiles,” to which modeled estimates of 7 conditions are added [36]. Until a consensus is reached about which chronic conditions and combinations of conditions should be monitored regionally or internationally, this study pleads for the assessment of a large but also reasoned list of conditions that independently impact mortality, disability, or perceived health. This requirement for an independent impact on health enables us to move beyond the debate about which type of condition (risk factors, symptoms, syndromes, diseases) should be considered in the multimorbidity assessment [37]: Any chronic or recurrent condition found in the current nosology (as reflected in the International Classification of Diseases, which includes diseases and sometimes syndromes and symptoms) with an independent impact on health should be considered as long as its frequency in the community justifies its monitoring. The magnitude of the impact may be considered among the prioritization criteria in order to limit the list.

### Multimorbid potential of conditions and meanings of the combinations

This study confirmed that several conditions have a high “multimorbid potential,” i.e., a high possibility of being associated with other conditions, which is notably the case with vasculometabolic and musculoskeletal conditions, chronic obstructive pulmonary disease, and especially mental disorders. These conditions were found in many dyads and triads in line with those retrieved by Prados-Torres and colleagues [9] and Violan and colleagues [8]. This suggests that interventions aimed at reducing the incidence of conditions with a high multimorbid potential could be an effective way to alleviate the burden of multimorbidity, which should thus be prioritized. On the contrary, as already reported [38], most cancers, multiple sclerosis, and dementia occur in a more isolated manner, although the underdiagnosis or underreporting of comorbid conditions cannot be excluded, in particular for dementia. One of the original aspects of this study, however, was the analysis of more than 200 dyads in terms of plausible etiological pathways. Using the typology of Valderas and colleagues [10], this analysis gave meaning to the combinations of conditions and provides clues for their clinical management beyond the simple mechanism of complication, which only drives a part of them. In particular, associations probably driven by bidirectional causality are especially important in the clinical setting if one condition is more treatable than another as in the case of depression and low back pain. Associations that share the same risk factors also require attention, as these factors may be more easily detected and tackled in multimorbid participants. In this regard, the major (and often causal) role played by obesity, depression, and anxiety in many associations, either as a component or as a shared risk factor of the components, should be stressed, especially as these conditions are not considered or are even excluded in several multimorbidity studies [39,40].

### Impact and joint effects of multimorbid associations

This study is one of the first to analytically address the impact and joint effects of a large number of multimorbid associations on activity limitations and perceived heath. These different but related aspects largely determine the potential severity of the multimorbid associations. As they directly reflect the burden experienced by the participants, the impact of multimorbid associations on health indicators is best investigated and reported in terms of absolute risks. Diseases with complications have the greatest impact on health, but associations of conditions that affect different systems also appear to have large impacts. Associations concerning systems involved in locomotion (cardiovascular, respiratory, osteoarticular), and to a lesser extent, those affecting sensorial functions strongly impact both activity limitations and perceived health, whereas associations with mental disorders mostly impact perceived health. The highly different impacts resulting from the association of 2, 3, or even 4 conditions, which form the bulk of the multimorbidity burden, make the simple counting of conditions inadequate in order to characterize the burden of multimorbidity. This inadequacy has recently been pointed out with regard to the impact on mortality [30,33,41].

The joint effects and especially the interactions of the components of associations, which are more closely related to biological mechanisms and synergy phenomena [42], probably deserve closer attention. In this study, interactions appeared to be extremely variable on a continuum from subadditive to supermultiplicative. The associations with multiplicative or supermultiplicative interactions, which included obesity, chronic obstructive pulmonary disease, migraine, and certain osteoarticular pathologies (inflammatory arthritis and osteoarthritis), were the most deleterious in terms of joint effects. By contrast, associations involving cardiometabolic conditions, low back pain, osteoporosis, injury sequelae, depression, and anxiety were less unfavorable for this effect. These results have important implications for multimorbidity measurements: The variable impacts and joint effects of associations would seem to preclude the exclusive use of a raw count of conditions to quantify multimorbidity.

### Strengths and limitations of the study

The strengths of this study include the use of 2 large and nationally representative surveys, including both cross-sectional and longitudinal data on 60 chronic or recurrent conditions and 6 health status measures, and the use of a multistep, systematic, and analytical approach to estimate the impact and the joint effects of conditions on health and to assess the underlying mechanisms explaining multimorbid associations. Moreover, the convergence and consistency of results across surveys, health indicators, and time frames further supports the robustness of our results, which was of particular importance due the type I statistical error that inevitably plagues the study of numerous morbidities.

This study also has some limitations. First, despite the relatively high levels of participation and follow-up rates achieved in the 2 nationwide surveys considered here, various marginal population groups (particularly severely ill participants) may have been underrepresented, thus limiting the external validity of the study. Second, self-reported information on chronic or recurrent conditions could be subject to an information bias due to social desirability (especially mental health conditions), age, and selective recall (symptom-based conditions are reported more frequently in surveys [43]). For many of the studied conditions, frequencies (1-year or lifetime frame) were similar to estimates from previous studies conducted in France except for obesity (when compared to studies where height and weight were measured [44]), and for conditions requiring institutionalization such as dementia or schizophrenia, which were less prevalent than expected. Underestimation of weight may represent a serious problem in terms of the control of confounding factors, since obesity is involved in many multimorbid associations. In other conditions, we presume that these biases mostly had a nondifferential effect on health indicators, possibly with the exception of the impact of disease severity on recall, as more severe conditions were more likely to be recalled and associated with poorer health status (severity was not directly assessed in this study, as in most others conducted to date on multimorbidity [21,45]). However, a lack of sensitivity in the reported information is preferable to a lack of specificity in terms of the nondifferential misclassification bias observed in association studies [46]. Third, the implementation of the approach to investigating the etiological pathways underlying multimorbid associations, using the typology proposed by Valderas and colleagues [10], suffers from several limitations: The empirical analyses used to classify into “chance only” and “confounding” categories may have been vulnerable to incomplete data (unrecorded conditions), to the cross-sectional nature of part of them, and sometimes to the lack of power; and the literature searching reflects the current knowledge, limited as regards causal associations between many conditions. Formal mediation analyses would have been preferable but were far from being achievable in, and also beyond the scope of, this study. Fourth, this study refrained from using data reduction methods such as factor or cluster analyses to investigate disease clustering into “patterns.” The implementation of these methods raises various problems, many of which still need to be resolved [47]. The consideration of disease combinations in dyads/triads/tetrads, as undertaken in this study, actually addressed patterns in three-quarters of multimorbid participants and also shed light on the process of morbidity aggregation (multimorbidity first involves two conditions, then three, then four, and so on). Fifth, unmeasured or incompletely controlled biological confounding cannot be excluded. Indeed, control for confounding is necessary when considering interactions [48]. The implications of this unquantifiable confounding would be primarily an attenuation of the associations. Finally, despite the large sample size, the power was limited to detecting small effects associated with less frequent conditions or even moderate effects in longitudinal analyses. The study of mortality was clearly underpowered due to the low number of deaths observed in the ESPS survey.

### Policy and research implications

The lack of methodological consensus or consistent approaches for studying multimorbidity has been a serious impediment for the development of appropriate control and prevention strategies. The proposed approach reported in this paper can guide future contributions with the aim to gain a better understanding and awareness of the different aspects of multimorbidity. Although undertaken in a single country (France), this study is replicable, as exemplified by the use of several survey datasets. It could therefore be readily implemented in many countries with existing systems for the surveillance of chronic diseases.

Multimorbidity is definitely not only a problem of old age; identification and monitoring should be implemented as early as midlife, possibly earlier in disadvantaged groups. Multimorbidity assessments should rapidly move beyond the simple counting of chronic conditions of any kind by including dozens of chronic conditions that impact health and taking into account variable impacts and joint effects of component conditions (e.g., monitoring the most frequent and impacting dyads or triads). Approaches to reduce the incidence of these combinations and minimize their impact should also be a priority for improving the health of multimorbid participants and reducing their impact on healthcare resources.

Further research is needed to evaluate the influence of the data collection method (self-reports, medical records, administrative data) and the comprehensiveness of data sources on medical conditions, which should definitely include obesity and mental disorders. Longitudinal studies of trajectories of multimorbid aggregations according to age, sex, and socioeconomic status and their impacts are also required, along with fundamental and clinical research on pathological interactions, notably between somatic and mental disorders. The clinical implications of the (statistical) concept of the interaction continuum from subadditive to supermultiplicative should be scrutinized. Without much delay, however, multimorbid combinations with large health impacts and most deleterious interactions should be appropriately monitored and managed within the healthcare system.

## Supporting information

S1 STROBE Checklist Checklist for Strengthening the Reporting of Observational Studies in Epidemiology (STROBE). (DOCX)

S1 Table Description of the studied samples (ESPS and HSM surveys). (DOCX)

S2 Table Associations of conditions with activity limitations, perceived health, or mortality in the ESPS and HSM surveys. (DOCX)

S3 Table Impact of studied chronic conditions on activity limitations and perceived health in the ESPS and HSM surveys. (DOCX)

S4 Table Risk of multimorbidity, defined as having at least 2 of the 48 selected conditions, associated with age, sex, and socioeconomic status indicators (education level, occupation, and household income) as estimated in multiple logistic regression models. (DOCX)

S5 Table Number of selected conditions and prevalence of multimorbidity according to age and sex. (DOCX)

S6 Table Risk of multimorbidity (vs monomorbidity) and number of conditions associated with each of the 48 chronic conditions retained. (DOCX)

S7 Table Weighted frequency of dyads of conditions, strength of associations, and plausible etiological pathways explaining the associations. (DOCX)

S8 Table Weighted frequency of triads and tetrads of conditions. (DOCX)

S9 Table Impact of associated conditions (dyads) on activity limitations and perceived health in the ESPS and HSM surveys. (DOCX)

S10 Table Impact of associated conditions (triads and tetrads) on activity limitations and perceived health in the ESPS and HSM surveys. (DOCX)

S11 Table Joint effects of dyads of associated conditions on activity limitations (severely limited in GALI, limited in ≥3 ADLs or ≥2 IADLs, new limitation) and perceived health (bad or very bad SRH, new health deterioration) in the ESPS and HSM surveys when the conditions were retained in the final models presented in Table 1. (DOCX)

S12 Table Joint effects of two-by-two associated conditions within triads on activity limitations and perceived health in the ESPS and HSM surveys when the conditions were retained in the final models presented in Table 1. (DOCX)

## Abbreviations

ADL: activities of daily living
AIC: Akaike information criterion
CI: confidence interval
COPD: chronic obstructive pulmonary disease
ESPS: Enquête Santé et Protection Sociale
GALI: Global Activity Limitation Indicator
GBD: Global Burden of Diseases
HSM: Enquête Handicap–Santé Ménages
IADL: instrumental activities of daily living
OR: odds ratio
RERI: relative excess risk due to interaction
RoOR: ratio of odds ratios
SF-12: Medical Outcomes Study Short-Form 12-Item Health Survey
SRH: Self-Reported Health
STROBE: Strengthening the Reporting of Observational Studies in Epidemiology
