# Supplementary material for: Estimating and characterizing the burden of multimorbidity in the community: A comprehensive multistep analysis of two large nationwide representative surveys in France
Source: PLoS Med. 2021 Apr 26;18(4):e1003584. doi: 10.1371/journal.pmed.1003584 (PMC8109815; doi:10.1371/journal.pmed.1003584)
Supplement: S1 STROBE Checklist — (DOCX) [file pmed.1003584.s001.docx]

S1 STROBE Checklist— Estimating and characterizing the burden of multimorbidity in the community: A comprehensive multi-step analysis of two large nationwide representative surveys in France

|  | | Item No | Recommendation | Page  No |
| --- | --- | --- | --- | --- |
| **Title and abstract** | | 1 | (*a*) Indicate the study’s design with a commonly used term in the title or the abstract | Title.  Abstract, “Methods and Findings” section, paragraph 1. |
|  |  |  | (*b*) Provide in the abstract an informative and balanced summary of what was done and what was found | Abstract “Methods and Findings” section, paragraphs 1 & 2. |
| Introduction | | | | |
| Background/rationale | | 2 | Explain the scientific background and rationale for the investigation being reported | Introduction, paragraph 1. |
| Objectives | | 3 | State specific objectives, including any prespecified hypotheses | Introduction, paragraph 2. |
| Methods | | | | |
| Study design | | 4 | Present key elements of study design early in the paper | Material and methods, “Survey designs and study populations” section, paragraph 1. |
| Setting | | 5 | Describe the setting, locations, and relevant dates, including periods of recruitment, exposure, follow-up, and data collection | Material and methods, “Survey designs and study populations” section, paragraphs 2 & 3. |
| Participants | | 6 | (*a*) *Cohort study*—Give the eligibility criteria, and the sources and methods of selection of participants. Describe methods of follow-up  *Case-control study*—Give the eligibility criteria, and the sources and methods of case ascertainment and control selection. Give the rationale for the choice of cases and controls  *Cross-sectional study*—Give the eligibility criteria, and the sources and methods of selection of participants | Material and methods, “Survey designs and study populations” section, paragraphs 2 & 3 |
|  |  |  | (*b*) *Cohort study*—For matched studies, give matching criteria and number of exposed and unexposed  *Case-control study*—For matched studies, give matching criteria and the number of controls per case | NA |
| Variables | | 7 | Clearly define all outcomes, exposures, predictors, potential confounders, and effect modifiers. Give diagnostic criteria, if applicable | Material and methods, “Chronic and recurrent conditions” and “Health status measures” sections. |
| Data sources/ measurement | | 8* | For each variable of interest, give sources of data and details of methods of assessment (measurement). Describe comparability of assessment methods if there is more than one group | Material and methods, “Chronic and recurrent conditions” and “Health status measures” sections. |
| Bias | | 9 | Describe any efforts to address potential sources of bias | Material and methods, “Survey designs and study populations”, paragraphs 1, 2 & 3. |
| Study size | | 10 | Explain how the study size was arrived at | Material and methods, “Survey designs and study populations” section, paragraphs 2 & 3. |
| Quantitative variables | | 11 | Explain how quantitative variables were handled in the analyses. If applicable, describe which groupings were chosen and why | Material and methods, “Statistical analysis” section, “Identifying the relevant conditions, associated with impacts on health status” subsection. |
| Statistical methods | | 12 | (*a*) Describe all statistical methods, including those used to control for confounding | Material and methods, “Statistical analysis” section. |
|  |  |  | (*b*) Describe any methods used to examine subgroups and interactions | Material and methods, “Statistical analysis” section, especially “Estimating the impact and joint effects of multimorbid associations” |
|  |  |  | (*c*) Explain how missing data were addressed | Material and methods, “Statistical analysis” section, 3^rd^ paragraph from end. |
|  |  |  | (*d*) *Cohort study*—If applicable, explain how loss to follow-up was addressed  *Case-control study*—If applicable, explain how matching of cases and controls was addressed  *Cross-sectional study*—If applicable, describe analytical methods taking account of sampling strategy | NA (Results section, 1^st^ paragraph indicates follow-up for 97% of the sample). |
|  |  |  | (*e*) Describe any sensitivity analyses | NA |
| Results | | | | |
| Participants | 13* | (a) Report numbers of individuals at each stage of study—eg numbers potentially eligible, examined for eligibility, confirmed eligible, included in the study, completing follow-up, and analysed | Material and methods, “Survey designs and study populations” section, paragraphs 2 & 3 & Results section, 1^st^ paragraph. | |
|  |  | (b) Give reasons for non-participation at each stage | Material and methods, “Survey designs and study populations” section, paragraphs 2 & 3 & Results section, 1^st^ paragraph. | |
|  |  | (c) Consider use of a flow diagram | The flow diagram does not seem necessary in this particular case. | |
| Descriptive data | 14* | (a) Give characteristics of study participants (eg demographic, clinical, social) and information on exposures and potential confounders | S1 Table. | |
|  |  | (b) Indicate number of participants with missing data for each variable of interest | Material and methods, “Statistical analysis” section, 3^rd^ paragraph from end. | |
|  |  | (c) *Cohort study*—Summarise follow-up time (eg, average and total amount) | Results section, 1^st^ paragraph. | |
| Outcome data | 15* | *Cohort study*—Report numbers of outcome events or summary measures over time | S1 Table. | |
|  |  | *Case-control study—*Report numbers in each exposure category, or summary measures of exposure |  | |
|  |  | *Cross-sectional study—*Report numbers of outcome events or summary measures | S1 Table. | |
| Main results | 16 | (*a*) Give unadjusted estimates and, if applicable, confounder-adjusted estimates and their precision (eg, 95% confidence interval). Make clear which confounders were adjusted for and why they were included | Table 3 & S2, S4, S6, S7, S11 & S12 Tables. | |
|  |  | (*b*) Report category boundaries when continuous variables were categorized | Material and methods, “Statistical analysis” section, “Identifying the relevant conditions, associated with impacts on health status” subsection. | |
|  |  | (*c*) If relevant, consider translating estimates of relative risk into absolute risk for a meaningful time period | NA. | |
| Other analyses | 17 | Report other analyses done—eg analyses of subgroups and interactions, and sensitivity analyses | Results, “Impact and joint effects of associations between conditions (dyads and triads)” section. | |
| Discussion | | | | |
| Key results | 18 | Summarise key results with reference to study objectives | Discussion, first paragraph. | |
| Limitations | 19 | Discuss limitations of the study, taking into account sources of potential bias or imprecision. Discuss both direction and magnitude of any potential bias | Discussion, “Strengths and limitations of the study” section. | |
| Interpretation | 20 | Give a cautious overall interpretation of results considering objectives, limitations, multiplicity of analyses, results from similar studies, and other relevant evidence | Discussion, “Strengths and limitations of the study” section. | |
| Generalisability | 21 | Discuss the generalisability (external validity) of the study results | Discussion, “Policy and research implications” section. | |
| Other information | | | | |
| Funding | 22 | Give the source of funding and the role of the funders for the present study and, if applicable, for the original study on which the present article is based | Funding information was entered in the financial disclosure section of the submission system. | |
