# Supplementary material for: Estimating and characterizing the burden of multimorbidity in the community: A comprehensive multistep analysis of two large nationwide representative surveys in France
Source: PLoS Med. 2021 Apr 26;18(4):e1003584. doi: 10.1371/journal.pmed.1003584 (PMC8109815; doi:10.1371/journal.pmed.1003584)
Supplement: S1 Table — (DOCX) [file pmed.1003584.s002.docx]

S1 Table. Description of the studied samples (ESPS and HSM surveys). All figures are weighted percentages unless otherwise indicated.

* Missing (questionnaire) in 9,808 subjects
** Follow-up was scheduled in 7,727 subjects
** Limitation (severe or not) in 2014 in subjects not limited in 2010 (N=2,101)
*** Health graded less than good in 2014 in subjects with good/very good health in 2010 (N=1886)
† Household incomes categorized according to tax bracket, split into three approximately equal parts (tertiles) in each survey. Subjects could refuse to provide their tax bracket

Abbreviations
ADL: activities of daily living; IADL: instrumental activities of daily living; SF-12: Medical Outcomes Study Short-Form 12-Item Health Survey: SEM: standard error of the mean
