# Supplementary material for: Estimating and characterizing the burden of multimorbidity in the community: A comprehensive multistep analysis of two large nationwide representative surveys in France
Source: PLoS Med. 2021 Apr 26;18(4):e1003584. doi: 10.1371/journal.pmed.1003584 (PMC8109815; doi:10.1371/journal.pmed.1003584)
Supplement: S2 Table — (DOCX) [file pmed.1003584.s003.docx]

S2 Table. Associations of conditions with activity limitations, perceived health, or mortality in the ESPS and HSM surveys. All estimates are weighted to represent the French general population estimates. The 48 conditions selected for further analysis are highlighted in bold.

|  | 1-year time frame (ESPS Survey) | | | | | | | | | | | Lifetime frame (HSM Survey) | | | | | | | | | |
| --- | --- | --- | --- | --- | --- | --- | --- | --- | --- | --- | --- | --- | --- | --- | --- | --- | --- | --- | --- | --- | --- |
|  | Cross-sectional analyses, ORs, and 95% CI for conditions included in final polytomous logistic models* | | | | | | Longitudinal analyses, ORs, and 95% CI for conditions included in final binary logistic models | | | | | Cross-sectional analyses, ORs, and 95% CI for conditions included in final polytomous logistic models* | | | | | | | | Cross-sectional analysis, regression coefficient, and 95% CI for conditions included in final linear regression model* | |
|  | GALI limited, severely | | GALI limited, not severely | | Bad or very bad health | Fair health | Death between 2010 and 2014 | | New limitation** | | New health deterioration*** | | GALI limited, severely | GALI limited, not severely | Bad or very bad health | Fair health | | Limitation in ≥ 3 ADLs or ≥ 2 IADLs | Limitation in < 3 ADLs and < 2 IADLs | | SF-12 total score |
| **HIV infection** |  |  | |  | |  | |  | |  |  | |  |  | 17.04  (2.11 – 137.38) | | 23.79  (2.80 – 202.16) |  |  | |  |
| **Colorectal cancer** | 3.72  (1.74 – 7.95) | 2.46  (1.21 – 5.02) | | 5.62  (2.29 – 13.80) | | 3.24  (1.51 – 6.95) | | 5.74  (2.46 – 13.39) | |  |  | | 3.21  (1.22 – 8.47) | 2.05  (0.89 – 4.72) | 5.30  (2.20 – 12.76) | | 2.80  (1.25 – 6.26) |  |  | | -9.85  (-16.69 – -3.01) |
| **Oropharyngeal and laryngeal cancer** | 10.81  (2.76 – 42.40) | 2.75  (0.60 – 12.56) | | 87.61  (8.99 – 853.64) | | 49.24  (6.86 – 353.62) | |  | |  |  | | 7.56  (2.78 – 20.60) | 6.13  (2.15 – 17.46) | 17.91  (5.90 – 54.35) | | 11.39  (3.79 – 34.18) | 4.09  (2.01 – 8.33) | 3.49  (1.54 – 7.90) | | -11.31  (-20.19 – -2.44) |
| **Lung cancer** | 42.21 (3.74 – 476.66) | 12.96 (1.25 – 134.86) | | 67.29  (4.52 – >999.999) | | 8.59  (0.63 – 117.89) | | 26.23  (8.07 – 85.33) | |  |  | | 24.48  (6.77 – 88.53) | 5.87  (1.78 – 19.33) | 35.14  (11.15 – 110.78) | | 12.94  (3.43 – 48.78) | 3.61  (1.71 – 7.63) | 3.12  (1.54 – 6.33) | | -18.23  (-26.83 – -9.63) |
| Skin cancer |  |  | |  | |  | |  | |  |  | |  |  |  | |  |  |  | |  |
| **Breast cancer** | 2.69  (1.23 – 5.85) | 3.56 (2.17 – 5.86) | | 3.69  (1.72 – 7.90) | | 2.79  (1.70 – 4.58) | | 5.47  (2.16 – 13.87) | |  |  | | 3.86  (2.46 – 6.06) | 3.96  (2.54 – 6.16) | 4.61  (2.76 – 7.71) | | 2.78  (1.82 – 4.25) | 1.20  (0.80 – 1.79) | 1.58  (1.06 – 2.38) | | -7.27  (-10.60 – -3.93) |
| Uterus cancer (endometrial and cervical) |  |  | |  | |  | |  | |  |  | |  |  | 2.68  (1.23 – 5.84) | | 1.92  (0.86 – 4.26) |  |  | |  |
| **Prostate cancer** |  |  | |  | |  | |  | |  |  | |  |  |  | |  |  |  | |  |
| **Urinary tract cancer (kidney, bladder)** |  |  | | 14.77  (3.61 – 60.36) | | 6.53  (1.76 – 24.19) | |  | |  |  | | 5.95  (1.94 – 18.26) | 3.25  (1.15 – 9.18) | 25.79  (5.96 – 111.63) | | 7.26  (1.70 – 30.88) |  |  | | -11.85  (-20.68 – -3.03) |
| **Other cancer** | 5.62  (2.45 – 12.87) | 2.80 (1.38 – 5.69) | | 8.42  (2.96 – 23.92) | | 4.96  (2.60 – 9.46) | | 5.47  (1.71 – 17.52) | |  |  | | 5.09  (3.15 – 8.24) | 2.17  (1.31 – 3.61) | 4.79  (2.75 – 8.37) | | 1.68  (1.00 – 2.84) | 2.73  (1.60 – 4.65) | 2.49  (1.46 – 4.25) | |  |
| **Malignant hemopathies** | 8.64  (3.34 – 22.31) | 3.34 (1.43 – 7.78) | | 6.45  (2.44 – 17.01) | | 1.33  (0.60 – 2.96) | |  | |  | 8.27  (1.08 – 63.51) | | 5.14  (2.43 – 10.85) | 2.51  (1.10 – 5.70) | 8.70  (3.59 – 21.06) | | 2.41  (1.05 – 5.54) | 2.55  (1.31 – 4.94) | 1.68  (0.80 – 3.53) | | -12.57  (-18.52 – -6.63) |
| Nutritional anemias | 6.85  (2.16 – 21.77) | 1.81  (0.43 – 7.70) | |  | |  | |  | |  |  | |  |  |  | |  |  |  | |  |
| Bleeding disorders |  |  | |  | |  | |  | |  |  | |  |  |  | |  |  |  | |  |
| **Thyroid disorders** |  |  | | 1.22  (0.84 – 1.77) | | 1.52  (1.19 – 1.93) | |  | |  |  | |  |  | 0.93  (0.73 – 1.19) | | 1.22  (1.00 – 1.51) |  |  | |  |
| **Diabetes** | 2.59  (1.91 – 3.52) | 2.04  (1.57 – 2.64) | | 5.68  (4.08 – 7.90) | | 2.67  (2.03 – 3.50) | | 1.83  (1.12 – 3.00) | |  |  | |  |  | 4.20  (3.31 – 5.32) | | 2.48  (1.99 – 3.10) | 1.70  (1.41 – 2.04) | 1.29  (1.06 – 1.57) | | -3.45  (-5.19 – -1.71) |
| **Obesity (morbid, BMI >35)†** | 4.99  (2.52 – 9.85) | 3.45  (1.98 – 5.99) | | 6.32  (3.06 – 13.07) | | 2.30  (1.22 – 4.34) | |  | |  |  | | 5.39  (3.51 – 8.30) | 2.19  (1.36 – 3.52) | 4.23  (2.44 – 7.35) | | 2.12  (1.18 – 3.81) | 3.59  (2.48 – 5.20) | 2.00  (1.34 – 2.98) | | -5.25  (-9.06 – -1.44) |
| **Obesity (nonmorbid, BMI 30-35)†** | 1.72  (1.34 – 2.22) | 1.35  (1.12 – 1.62) | | 1.70  (1.31 – 2.21) | | 1.47  (1.24 – 1.76) | |  | | 1.68  (1.15 – 2.46) | 1.92  (1.29 – 2.86) | | 2.08  (1.76 – 2.46) | 1.59  (1.37 – 1.86) | 1.99  (1.66 – 2.40) | | 1.58  (1.36 – 1.84) | 1.36  (1.15 – 1.59) | 1.69  (1.42 – 2.01) | | -3.27  (-4.42 – -2.11) |
| Metabolic abnormalities (hyperlipidemia) |  |  | |  | |  | |  | |  |  | |  |  |  | |  |  |  | |  |
| **Substance abuse** |  |  | |  | |  | | 9.30  (1.42 – 60.73) | |  |  | | 18.11  (3.70 – 88.64) | 7.16  (1.23 – 41.73) | 29.57  (4.26 – 205.48) | | 7.32  (1.44 – 37.11) | 2.25  (0.43 – 11.84) | 17.38  (4.29 – 70.40) | | -15.16  (-24.01 – -6.30) |
| **Schizophrenia** | 16.31  (1.35 – 196.82) | 5.14  (0.35 – 74.72) | |  | |  | |  | |  |  | | 34.17  (13.60 – 85.88) | 6.57  (2.44 – 17.69) | 14.03  (6.17 – 31.91) | | 4.90  (2.13 – 11.27) | 63.81  (29.29 – 139.04) | 20.60  (9.68 – 43.86) | | -19.73  (-25.44 – -14.01) |
| **Depression** | 3.24  (2.35 – 4.47) | 1.90  (1.46 – 2.47) | | 5.05  (3.66 – 6.96) | | 2.30  (1.79 – 2.94) | |  | |  |  | | 6.00  (4.49 – 8.01) | 3.22  (2.42 – 4.28) | 8.36  (5.91 – 11.82) | | 3.27  (2.39 – 4.47) | 2.76  (2.16 – 3.54) | 2.40  (1.90 – 3.05) | | -14.32  (-16.36 – -12.27) |
| **Anxiety** | 1.55  (1.18 – 2.05) | 1.27  (1.04 – 1.57) | | 2.44  (1.86 – 3.20) | | 1.81  (1.50 – 2.17) | |  | | 22.96  (1.59 – 330.77) | | | 1.86  (1.46 – 2.36) | 1.41  (1.13 – 1.76) | 2.87  (2.19 – 3.76) | | 2.35  (1.82 – 3.04) | 1.85  (1.46 – 2.33) | 1.48  (1.19 – 1.86) | | -8.18  (-9.95 – -6.41) |

Abbreviations. OR: odds ratio; CI: confidence interval; BMI: body mass index; HIV: human immunodeficiency virus; GALI: Global Activity Limitation Indicator; SRH: Self-Reported Health indicator; ADL: limitation in activity of daily living; IADL: limitation in instrumental activity of daily living

* Polytomous logistic regression using “no limitation” or “good or very good heath” or “no limitation in ADL/IADL” as the reference category. All models include age and sex
** Limitation, severe or not in 2014 in subjects who were not limited in 2010
*** Health graded less than good in 2014 in subjects with good/very good health in 2010
† Obesity was categorized according to the standard BMI criteria (obese: BMI 30–35; morbidly obese: BMI > 35) and analyzed as a three-category variable
†† Odds ratio or regression coefficient associated with 1-point (1-condition) increase. Dose–response analyses with restricted cubic splines indicate changes in slope (negative) at 3 and 6 conditions except for SF-12 where the change in slope is observed at 4 conditions.

S2 Table (continued). Associations of conditions with activity limitations, perceived health, or mortality in the ESPS and HSM surveys. All estimates are weighted to represent the French general population estimates. The 47 conditions selected for further analysis are highlighted in bold.

|  | 1-year time frame (ESPS Survey) | | | | | | | | | | | Lifetime frame (HSM Survey) | | | | | | | | | |
| --- | --- | --- | --- | --- | --- | --- | --- | --- | --- | --- | --- | --- | --- | --- | --- | --- | --- | --- | --- | --- | --- |
|  | Cross-sectional analyses, ORs, and 95% CI for conditions included in final polytomous logistic models* | | | | | | Longitudinal analyses, ORs, and 95% CI for conditions included in final binary logistic models | | | | | Cross-sectional analyses, ORs, and 95% CI for conditions included in final polytomous logistic models* | | | | | | | | Cross-sectional analysis, regression coefficient, and 95% CI for conditions included in final linear regression model* | |
|  | GALI limited, severely | | GALI limited, not severely | | Bad or very bad health | Fair health | Death between 2010 and 2014 | | New limitation** | | New health deterioration*** | | GALI limited, severely | GALI limited, not severely | Bad or very bad health | Fair health | | Limitation in ≥ 3 ADLs or ≥ 2 IADLs | Limitation in < 3 ADLs and < 2 IADLs | | SF-12 total score |
| **Parkinson’s disease** | 7.86  (2.47 – 25.04) | 3.15  (0.97 – 10.25) | | 14.08  (4.03 – 49.20) | | 2.33  (0.64 – 8.54) | |  | |  |  | | 20.85  (5.06 – 85.87) | 5.14  (1.36 – 19.41) | 14.48  (3.06 – 68.59) | | 3.71  (1.00 – 13.77) | 16.27  (5.77 – 45.88) | 3.29  (1.33 – 8.15) | | -19.34  (-23.72 – -14.95) |
| **Alzheimer's disease and other dementias** | 16.50  (5.09 – 53.51) | 3.62 (1.13 – 11.58) | | 23.05  (4.93 – 107.72) | | 5.08  (1.37 – 18.88) | | 5.87  (0.97 – 35.68) | |  |  | | 19.67  (9.63 – 40.16) | 3.90  (1.84 – 8.28) | 9.10  (4.89 – 16.95) | | 3.01  (1.63 – 5.55) | 55.30  (26.12 – 117.08) | 6.68  (2.61 – 17.11) | | -22.62  (-28.04 – -17.21) |
| **Multiple sclerosis** | 164.80  (20.30 – >1000) | 59.97  (6.46 – 557.04) | | 203.19  (38.36 – >1000) | | 18.43  (2.43 – 139.79) | |  | |  |  | | 66.73  (30.25 – 147.17) | 16.16  (6.78 – 38.50) | 56.60  (25.93 – 123.51) | | 24.68  (11.88 – 51.26) | 32.26  (13.36 – 77.88) | 3.99  (1.71 – 9.33) | | -21.80  (-28.79 – -14.81) |
| **Epilepsy** | 2.33  (1.02 – 5.31) | 1.89  (0.87 – 4.10) | | 4.96  (2.07 – 11.88) | | 2.29  (1.09 – 4.83) | |  | |  |  | | 5.74  (3.54 – 9.30) | 2.36  (1.46 – 3.80) | 5.22  (3.23 – 8.44) | | 2.56  (1.67 – 3.94) | 6.90  (4.00 – 11.88) | 3.55  (2.25 – 5.61) | |  |
| **Migraine** | 1.01  (0.71 – 1.43) | 1.63  (1.31 – 2.03) | | 2.09  (1.54 – 2.84) | | 1.64  (1.34 – 2.01) | |  | |  |  | | 1.32  (1.10 – 1.60) | 1.27  (1.05 – 1.53) | 1.69  (1.37 – 2.09) | | 1.35  (1.11 – 1.64) | 1.60  (1.27 – 2.00) | 1.32  (1.08 – 1.61) | | -3.72  (-5.10 – -2.34) |
| **Cataract** |  |  | |  | |  | |  | |  |  | |  |  |  | |  | 1.29  (1.05 – 1.58) | 1.32  (1.06 – 1.65) | |  |
| **Iris, choroidal, and retinal diseases** | 2.38  (1.08 – 5.22) | 1.98  (1.01 – 3.85) | | 2.95  (1.29 – 6.78) | | 1.76  (0.97 – 3.17) | |  | |  |  | | 3.20  (1.83 – 5.59) | 2.66  (1.49 – 4.75) |  | |  | 3.08  (2.04 – 4.66) | 1.75  (1.10 – 2.77) | |  |
| **Glaucoma** |  |  | |  | |  | |  | |  | 2.29  (1.06 – 4.95) | | 2.03  (1.41 – 2.94) | 1.98  (1.33 – 2.94) |  | |  | 1.50  (1.09 – 2.07) | 1.05  (0.71 – 1.56) | | -3.75  (-7.31 – -0.19) |
| **Ear ailments** | 1.41  (1.08 – 1.85) | 1.46  (1.19 – 1.79) | | 1.38  (1.05 – 1.81) | | 1.48  (1.22 – 1.78) | |  | |  |  | |  |  | 1.31  (1.02 – 1.69) | | 1.31  (1.06 – 1.62) |  |  | |  |
| **Hypertension** |  |  | | 1.31  (1.03 – 1.66) | | 1.49  (1.28 – 1.74) | |  | |  |  | | 0.94  (0.81 – 1.09) | 1.26  (1.09 – 1.45) | 1.34  (1.14 – 1.58) | | 1.50  (1.29 – 1.74) |  |  | |  |
| **Ischemic heart disease** | 1.62  (0.95 – 2.74) | 1.85  (1.19 – 2.87) | | 1.84  (1.01 – 3.37) | | 1.91  (1.17 – 3.13) | |  | |  |  | | 4.30  (3.02 – 6.13) | 3.42  (2.44 – 4.81) | 4.02  (2.66 – 6.08) | | 2.51  (1.74 – 3.62) | 1.58  (1.22 – 2.05) | 1.46  (1.10 – 1.95) | | -7.64  (-10.20 – -5.08) |
| **Myocardial infarction** | 4.61  (2.49 – 8.56) | 2.50 (1.41 – 4.44) | | 3.88  (1.99 – 7.58) | | 1.74  (0.97 – 3.13) | |  | |  |  | | 3.37  (2.15 – 5.27) | 2.40  (1.60 – 3.59) | 3.09  (1.99 – 4.82) | | 2.22  (1.48 – 3.34) |  |  | | -3.75  (-6.79 – -0.71) |
| **Cardiac rhythm disorders** | 2.95  (2.18 – 3.98) | 1.94 (1.49 – 2.52) | | 3.58  (2.55 – 5.04) | | 2.46  (1.88 – 3.22) | |  | |  |  | | 1.67  (1.24 – 2.25) | 1.16  (0.86 – 1.55) | 2.77  (1.99 – 3.85) | | 2.11  (1.54 – 2.90) |  |  | | -3.00  (-5.32 – -0.68) |
| **Heart failure** |  |  | |  | |  | |  | |  |  | | 5.84  (3.77 – 9.05) | 4.14  (2.72 – 6.31) | 7.30  (4.64 – 11.49) | | 3.20  (2.14 – 4.80) | 2.31  (1.77 – 3.02) | 1.76  (1.31 – 2.36) | | -6.29  (-8.95 – -3.63) |
| **Stroke** | 4.60  (2.49 – 8.50) | 1.98  (1.15 – 3.39) | | 6.71  (3.59 – 12.56) | | 3.14  (1.85 – 5.33) | |  | |  |  | | 6.61  (4.34 – 10.05) | 2.94  (1.92 – 4.50) | 4.18  (2.74 – 6.37) | | 2.56  (1.72 – 3.81) | 4.69  (3.42 – 6.45) | 2.81  (1.95 – 4.03) | | -8.36  (-11.52 – -5.19) |
| **Peripheral arterial disease** | 3.05  (1.66 – 5.59) | 1.44  (0.83 – 2.49) | | 2.38  (1.19 – 4.75) | | 1.77  (0.98 – 3.19) | |  | |  |  | | 5.41  (2.87 – 10.19) | 3.67  (1.99 – 6.74) | 5.38  (3.03 – 9.55) | | 3.44  (1.93 – 6.13) |  |  | | -7.36  (-11.23 – -3.49) |
| Lower extremity varices |  |  | |  | |  | |  | |  |  | |  |  |  | |  |  |  | |  |
| Hemorrhoids |  |  | |  | |  | |  | |  |  | |  |  |  | |  |  |  | |  |
| Allergic rhinitis |  |  | |  | |  | |  | |  |  | |  |  |  | |  |  |  | |  |
| **Chronic obstructive pulmonary disease** | 2.01  (1.27 – 3.19) | 1.41  (0.96 – 2.06) | | 3.17  (1.98 – 5.07) | | 2.04  (1.39 – 2.99) | |  | | 2.44  (1.07 – 5.57) | 3.01  (1.10 – 8.21) | | 2.21  (1.74 – 2.82) | 1.73  (1.35 – 2.22) | 2.28  (1.73 – 3.01) | | 1.77  (1.37 – 2.27) | 1.76  (1.39 – 2.22) | 1.39  (1.11 – 1.75) | | -5.40  (-7.31 – -3.48) |
| **Asthma** | 2.21  (1.49 – 3.27) | 1.85  (1.35 – 2.54) | | 1.86  (1.24 – 2.80) | | 1.62  (1.23 – 2.13) | |  | |  |  | | 1.83  (1.44 – 2.32) | 1.72  (1.36 – 2.17) | 1.54  (1.21 – 1.96) | | 1.66  (1.33 – 2.06) | 1.22  (0.96 – 1.54) | 1.34  (1.06 – 1.71) | | -3.75  (-5.69 – -1.81) |
| **Peptic ulcer** | 2.27  (1.37 – 3.76) | 1.34  (0.90 – 2.01) | | 3.62  (2.21 – 5.94) | | 2.14  (1.47 – 3.11) | |  | |  |  | | 1.59  (1.15 – 2.19) | 1.24  (0.91 – 1.69) | 2.57  (1.81 – 3.64) | | 1.77  (1.29 – 2.42) | 1.29  (1.01 – 1.65) | 1.64  (1.26 – 2.12) | | -2.79  (-4.85 – -0.73) |
| **Inflammatory bowel diseases** |  |  | |  | |  | |  | |  |  | | 3.04  (1.61 – 5.75) | 2.74  (1.27 – 5.90) | 5.43  (2.19 – 13.51) | | 1.67  (0.86 – 3.24) |  |  | |  |
| Food allergies |  |  | |  | |  | |  | |  |  | |  |  |  | |  |  |  | |  |
| **Chronic liver diseases** | 3.15  (1.39 – 7.12) | 2.47  (1.14 – 5.37) | | 6.76  (2.48 – 18.46) | | 2.78  (1.31 – 5.89) | | 6.22  (2.47 – 15.65) | |  |  | | 2.71  (1.57 – 4.67) | 1.92  (1.07 – 3.42) | 4.33  (2.12 – 8.83) | | 2.64  (1.26 – 5.53) |  |  | | -7.32  (-11.40 – -3.24) |

Abbreviations. OR: odds ratio; CI: confidence interval; BMI: body mass index; HIV: human immunodeficiency virus; GALI: Global Activity Limitation Indicator; SRH: Self-Reported Health indicator; ADL: limitation in activity of daily living; IADL: limitation in instrumental activity of daily living

* Polytomous logistic regression using “no limitation” or “good or very good heath” or “no limitation in ADL/IADL” as the reference category. All models include age and sex
** Limitation, severe or not in 2014 in subjects who were not limited in 2010
*** Health graded less than good in 2014 in subjects with good/very good health in 2010
† Obesity was categorized according to the standard BMI criteria (obese: BMI 30–35; morbidly obese: BMI > 35) and analyzed as a three-category variable
†† Odds ratio or regression coefficient associated with 1-point (1-condition) increase. Dose–response analyses with restricted cubic splines indicate changes in slope (negative) at 3 and 6 conditions except for SF-12 where the change in slope is observed at 4 conditions.

S2 Table (continued). Associations of conditions with activity limitations, perceived health, or mortality in the ESPS and HSM surveys. All estimates are weighted to represent the French general population estimates. The 47 conditions selected for further analysis are highlighted in bold.

|  | 1-year time frame (ESPS Survey) | | | | | | | | | | | Lifetime frame (HSM Survey) | | | | | | | | | |
| --- | --- | --- | --- | --- | --- | --- | --- | --- | --- | --- | --- | --- | --- | --- | --- | --- | --- | --- | --- | --- | --- |
|  | Cross-sectional analyses, ORs, and 95% CI for conditions included in final polytomous logistic models* | | | | | | Longitudinal analyses, ORs, and 95% CI for conditions included in final binary logistic models | | | | | Cross-sectional analyses, ORs, and 95% CI for conditions included in final polytomous logistic models* | | | | | | | | Cross-sectional analysis, regression coefficient, and 95% CI for conditions included in final linear regression model* | |
|  | GALI limited, severely | | GALI limited, not severely | | Bad or very bad health | Fair health | Death between 2010 and 2014 | | New limitation** | | New health deterioration*** | | GALI limited, severely | GALI limited, not severely | Bad or very bad health | Fair health | | Limitation in ≥ 3 ADLs or ≥ 2 IADLs | Limitation in < 3 ADLs and < 2 IADLs | | SF-12 total score |
| Eczema |  |  | |  | |  | |  | |  |  | |  |  |  | |  |  |  | |  |
| Psoriasis |  |  | |  | |  | |  | |  |  | |  |  |  | |  |  |  | |  |
| **Rheumatoid arthritis** | 7.58  (2.32 – 24.83) | 7.00  (2.40 – 20.44) | | 5.14  (1.39 – 19.05) | | 2.96  (1.01 – 8.67) | |  | |  | 17.25  (1.27 – 234.47) | | 5.85  (3.98 – 8.59) | 3.97  (2.67 – 5.91) | 7.17  (4.59 – 11.20) | | 2.99  (1.95 – 4.58) | 2.02  (1.50 – 2.71) | 1.77  (1.28 – 2.45) | | -7.05  (-10.09 – -4.01) |
| **Other (non-rheumatoid) inflammatory arthritis** | 6.78  (2.95 – 15.58) | 2.87  (1.49 – 5.53) | | 8.40  (3.92 – 18.01) | | 3.46  (1.94 – 6.18) | |  | |  |  | | 3.13  (2.27 – 4.31) | 2.55  (1.89 – 3.45) | 3.16  (2.26 – 4.41) | | 2.16  (1.61 – 2.89) | 1.65  (1.25 – 2.20) | 1.64  (1.27 – 2.11) | | -5.29  (-7.54 – -3.04) |
| **Osteoarthritis of the hip** | 2.42  (1.70 – 3.43) | 1.72  (1.28 – 2.32) | | 2.22  (1.54 – 3.22) | | 1.45  (1.08 – 1.96) | |  | |  |  | | 2.48  (1.94 – 3.18) | 1.90  (1.50 – 2.41) | 1.99  (1.53 – 2.57) | | 1.60  (1.26 – 2.04) | 1.26  (1.03 – 1.54) | 1.40  (1.13 – 1.74) | | -5.16  (-7.02 – -3.31) |
| **Osteoarthritis of the knee** | 1.72  (1.30 – 2.26) | 1.83  (1.49 – 2.26) | | 1.78  (1.34 – 2.36) | | 1.54  (1.25 – 1.90) | |  | | 2.01  (1.30 – 3.12) | 2.26  (1.43 – 3.57) | | 2.44  (2.01 – 2.97) | 2.39  (1.98 – 2.88) | 2.21  (1.80 – 2.72) | | 1.93  (1.60 – 2.34) | 1.37  (1.15 – 1.62) | 1.16  (0.97 – 1.38) | | -6.34  (-7.88 – -4.79) |
| **Osteoarthritis of other peripheral joints** | 2.23  (1.70 – 2.92) | 2.09  (1.69 – 2.58) | | 2.63  (2.01 – 3.45) | | 1.61  (1.32 – 1.97) | |  | | 1.79  (1.16 – 2.75) | 1.69  (1.04 – 2.73) | | 2.61  (2.19 – 3.11) | 2.07  (1.75 – 2.44) | 2.45  (2.05 – 2.93) | | 2.04  (1.72 – 2.41) | 1.24  (1.06 – 1.45) | 1.44  (1.21 – 1.71) | | -4.99  (-6.38 – -3.61) |
| **Low back pain** | 1.54  (1.23 – 1.95) | 2.16  (1.84 – 2.54) | | 1.80  (1.44 – 2.26) | | 1.72  (1.48 – 1.99) | |  | | 2.04  (1.45 – 2.85) | 1.69  (1.15 – 2.49) | | 1.79  (1.53 – 2.08) | 2.11  (1.83 – 2.43) | 1.91  (1.64 – 2.24) | | 1.94  (1.69 – 2.24) | 1.07  (0.91 – 1.25) | 1.32  (1.13 – 1.54) | | -2.93  (-3.94 – -1.91) |
| **Osteoporosis** | 1.62  (1.08 – 2.43) | 0.94  (0.67 – 1.31) | | 1.89  (1.21 – 2.95) | | 1.51  (1.10 – 2.07) | |  | |  |  | | 1.34  (1.00 – 1.82) | 1.03  (0.75 – 1.41) | 1.95  (1.43 – 2.68) | | 1.52  (1.13 – 2.05) |  |  | | -2.78  (-5.08 – -0.48) |
| **Kidney failure** | 15.64  (2.37 – 103.07) | 8.86  (1.26 – 62.13) | |  | |  | |  | |  |  | | 11.10  (4.33 – 28.45) | 2.90  (1.03 – 8.14) | 11.11  (3.75 – 32.92) | | 3.07  (0.93 – 10.08) | 5.04  (1.82 – 13.94) | 3.49  (1.26 – 9.66) | |  |
| Repeated urinary infections |  |  | |  | |  | |  | |  |  | |  |  |  | |  |  |  | |  |
| **Urinary incontinence** | 9.41  (3.66 – 24.20) | 4.99  (1.82 – 13.69) | | 105.10  (8.42 – >1000) | | 24.80  (2.01 – 305.79) | |  | |  |  | | 2.66  (1.93 – 3.68) | 1.46  (1.03 – 2.07) | 2.35  (1.66 – 3.32) | | 1.46  (1.03 – 2.08) | 2.52  (1.92 – 3.30) | 1.26  (0.96 – 1.65) | | -3.34  (-5.63 – -1.05) |
| Benign prostatic hypertrophy |  |  | |  | |  | |  | |  |  | |  |  |  | |  |  |  | |  |
| **Injury sequelae** | 13.36  (7.68 – 23.27) | 5.90  (3.47 – 10.03) | | 4.72  (2.80 – 7.94) | | 1.78  (1.16 – 2.72) | |  | | 3.65  (1.21 – 11.01) | 7.56  (2.99 – 19.09) | | 3.08  (2.39 – 3.96) | 2.55  (2.03 – 3.22) | 1.85  (1.42 – 2.40) | | 1.58  (1.26 – 1.99) | 1.79  (1.39 – 2.30) | 1.99  (1.58 – 2.51) | | -5.34  (-7.10 – -3.57) |
| Raw count of the 48 selected conditions†† | 1.85  (1.77 – 1.94) | 1.59 (1.53 – 1.65) | | 2.21 (2.10 – 2.33) | | 1.68 (1.61 – 1.75) | | 1.08 (0.99 – 1.16) | | 1.38 (1.27 – 1.50) | 1.41 (1.28 – 1.56) | | 2.02 (1.95 – 2.10) | 1.72 (1.66 – 1.78) | 2.27 (2.18 – 2.36) | | 1.77 (1.71 – 1.84) | 1.47 (1.44 – 1.51) | 1.39 (1.36 – 1.43) | | -4,50 (-4.72 – -4.29) |

Abbreviations. OR: odds ratio; CI: confidence interval; BMI: body mass index; HIV: human immunodeficiency virus; GALI: Global Activity Limitation Indicator; SRH: Self-Reported Health indicator; ADL: limitation in activity of daily living; IADL: limitation in instrumental activity of daily living

* Polytomous logistic regression using “no limitation” or “good or very good heath” or “no limitation in ADL/IADL” as the reference category. All models include age and sex
** Limitation, severe or not in 2014 in subjects who were not limited in 2010
*** Health graded less than good in 2014 in subjects with good/very good health in 2010
† Obesity was categorized according to the standard BMI criteria (obese: BMI 30–35; morbidly obese: BMI > 35) and analyzed as a three-category variable
†† Odds ratio or regression coefficient associated with 1-point (1-condition) increase. Dose–response analyses with restricted cubic splines indicate changes in slope (negative) at 3 and 6 conditions except for SF-12 where the change in slope is observed at 4 conditions.
