# Supplementary material for: Estimating and characterizing the burden of multimorbidity in the community: A comprehensive multistep analysis of two large nationwide representative surveys in France
Source: PLoS Med. 2021 Apr 26;18(4):e1003584. doi: 10.1371/journal.pmed.1003584 (PMC8109815; doi:10.1371/journal.pmed.1003584)
Supplement: S3 Table — (DOCX) [file pmed.1003584.s004.docx]

S3 Table. Impact of studied chronic conditions on activity limitations and perceived health in the ESPS and HSM surveys. Figures represent weighted percentages of subjects presenting limitations or deteriorated perceived health at the indicated threshold.

Abbreviations

BMI: body mass index; HIV: human immunodeficiency virus; GALI: Global Activity Limitation Indicator; SRH: Self-Reported Health indicator; ADL: limitation in activity of daily living: IADL: limitation in instrumental activity of daily living
