# Supplementary material for: Estimating and characterizing the burden of multimorbidity in the community: A comprehensive multistep analysis of two large nationwide representative surveys in France
Source: PLoS Med. 2021 Apr 26;18(4):e1003584. doi: 10.1371/journal.pmed.1003584 (PMC8109815; doi:10.1371/journal.pmed.1003584)
Supplement: S4 Table — (DOCX) [file pmed.1003584.s005.docx]

S4 Table. Risk of multimorbidity, defined as having at least two of the 48 selected conditions, associated with age, sex, and socioeconomic status indicators (education level, occupation, and household income) as estimated in multiple logistic regression models. Conditions are based on their presence during the last 12 months (ESPS Survey) or their lifetime occurrence (HSM Survey). Adjusted odds ratios and 95% confidence intervals are shown.
