# Supplementary material for: Estimating and characterizing the burden of multimorbidity in the community: A comprehensive multistep analysis of two large nationwide representative surveys in France
Source: PLoS Med. 2021 Apr 26;18(4):e1003584. doi: 10.1371/journal.pmed.1003584 (PMC8109815; doi:10.1371/journal.pmed.1003584)
Supplement: S5 Table — (DOCX) [file pmed.1003584.s006.docx]

S5 Table. Number of selected conditions and prevalence of multimorbidity according to age and sex. Figures represent weighted percentages of subjects with the corresponding number of conditions. Conditions are present during the last 12 months (ESPS Survey) and lifetime (HSM Survey).
