# Supplementary material for: Estimating and characterizing the burden of multimorbidity in the community: A comprehensive multistep analysis of two large nationwide representative surveys in France
Source: PLoS Med. 2021 Apr 26;18(4):e1003584. doi: 10.1371/journal.pmed.1003584 (PMC8109815; doi:10.1371/journal.pmed.1003584)
Supplement: S6 Table — (DOCX) [file pmed.1003584.s007.docx]

S6 Table. Risk of multimorbidity (vs monomorbidity) and number of conditions associated with each of the 48 chronic conditions retained.

* Risk adjusted on age and sex, odds ratios, and 95% confidence intervals
** Obesity was categorized according to the standard BMI criteria (obese: BMI 30–35; morbidly obese: BMI > 35) and analyzed as a three-category variable

Abbreviations
BMI: body mass index; HIV: human immunodeficiency virus
