# Supplementary material for: Estimating and characterizing the burden of multimorbidity in the community: A comprehensive multistep analysis of two large nationwide representative surveys in France
Source: PLoS Med. 2021 Apr 26;18(4):e1003584. doi: 10.1371/journal.pmed.1003584 (PMC8109815; doi:10.1371/journal.pmed.1003584)
Supplement: S7 Table — (DOCX) [file pmed.1003584.s008.docx]

S7 Table. Weighted frequency of dyads of conditions, strength of associations, and plausible etiological pathways explaining the associations. All dyads with a frequency of ≥ 0.25% in at least one survey sample are considered. Dyads are presented in decreasing order of frequency (mean frequency based on the two surveys).

|  | 1-year time frame (ESPS Survey) | | | Lifetime frame (HSM Survey) | | |  |
| --- | --- | --- | --- | --- | --- | --- | --- |
| Dyad | Frequency | Age- and sex-adjusted OR | Fully adjusted* OR | Frequency | Age- and sex-adjusted OR | Fully adjusted* OR | Plausible etiological pathway(s) explaining the association |
| Hypertension-Low back pain | 3.63 | 2.23 (1.94-2.58) | 1.60 (1.36-1.88) | 3.73 | 1.53 (1.36-1.72) | 1.44 (1.27-1.63) | Shared determinants (obesity, smoking, mental) |
| Obesity, nonmorbid-Hypertension | 3.40 | 4.22 (3.61-4.93) | 2.98 (2.50-3.56) | 3.50 | 2.49 (2.18-2.84) | 2.24 (1.95-2.58) | Causal (determinant) |
| Hypertension-Osteoarthritis of the knee | 3.56 | 2.65 (2.25-3.12) | 1.61 (1.33-1.94) | 2.41 | 1.38 (1.20-1.59) | 1.09 (0.95-1.27) | Shared determinants (obesity, mental) |
| Osteoarthritis of the knee-Low back pain | 3.03 | 4.03 (3.44-4.72) | 2.63 (2.21-3.14) | 2.88 | 2.42 (2.12-2.76) | 1.67 (1.44-1.93) | Shared determinants (obesity, mental) |
| Migraine-Low back pain | 2.08 | 4.03 (3.40-4.76) | 2.45 (2.02-2.99) | 3.37 | 3.11 (2.70-3.57) | 2.41 (2.07-2.81) | Shared determinants (obesity, mental) |
| Osteoarthritis of other peripheral joints-Low back pain | 2.30 | 2.99 ( 2.52-3.55) | 2.59 (2.16-3.11) | 3.15 | 2.10 (1.84-2.40) | 1.52 (1.31-1.76) | Shared determinants (obesity, mental) |
| Hypertension-Osteoarthritis of other peripheral joints | 2.74 | 2.32 (1.94-2.78) | 2.02 (1.67-2.44) | 2.56 | 1.24 (1.08-1.42) | 1.13 (0.98-1.30) | Shared determinants (obesity, mental) |
| Anxiety-Low back pain | 3.04 | 3.93 (3.39-4.56) | 2.52 (2.12-3.00) | 2.17 | 3.14 (2.69-3.67) | 2.25 (1.91-2.65) | Causal (bidirectional) |
| Diabetes-Hypertension | 2.67 | 5.53 (4.46-6.85) | 3.99 (3.19-4.98) | 2.42 | 2.75 (2.35-3.21) | 2.13 (1.82-2.51) | Shared determinants (obesity) |
| Osteoarthritis of the knee-Osteoarthritis of other peripheral joints | 2.11 | 3.57 (2.94-4.33) | 2.08 (1.67-2.60) | 2.50 | 2.83 (2.43-3.29) | 2.02 (1.72-2.38) | Strongly related pathological processes |
| Osteoarthritis of the hip-Osteoarthritis of the knee | 2.31 | 11.23 (8.99-14.02) | 6.48 (5.10-8.25) | 2.10 | 5.85 (4.94-6.92) | 4.27 (3.60-5.06) | Strongly related pathological processes |
| Obesity, nonmorbid-Low back pain | 2.04 | 1.98 (1.70-2.32) | 1.33 (1.11-1.60) | 2.35 | 1.04 (0.91-1.18) | - | Causal (determinant) |
| Ear ailments-Hypertension | 3.02 | 2.08 (1.76-2.47) | 1.36 (1.12-1.65) | 1.16 | 1.30 (1.07-1.57) | 1.19 (0.98-1.44) | Shared determinants (mental) |
| Depression-Anxiety | 2.69 | 12.87 (10.65-15.56) | 9.20 (7.45-11.37) | 1.45 | 11.07 (9.13-13.42) | 8.16 (6.61-10.06) | Strongly related pathological processes |
| Obesity, nonmorbid-Osteoarthritis of the knee | 1.97 | 3.49 (2.91-4.17) | 2.33 (1.89-2.87) | 2.09 | 2.48 (2.15-2.86) | 2.43 (2.10-2.81) | Causal (bidirectional) |
| Ear ailments-Low back pain | 2.50 | 2.93 (2.49-3.45) | 2.00 (1.68-2.40) | 1.49 | 1.77 (1.49-2.11) | 1.44 (1.19-1.74) | Shared determinants (mental) |
| Osteoarthritis of the hip-Low back pain | 1.81 | 4.62 (3.78-5.66) | 2.24 (1.76-2.86) | 2.09 | 3.03 (2.57-3.56) | 2.16 (1.81-2.57) | Shared determinants (obesity, mental) |
| Anxiety-Hypertension | 2.51 | 2.17 (1.82-2.60) | 1.57 (1.29-1.92) | 1.35 | 1.61 (1.37-1.91) | 1.39 (1.17-1.64) | Causal (bidirectional) |
| Migraine-Anxiety | 1.95 | 5.11 (4.26-6.13) | 3.43 (2.81-4.18) | 1.60 | 4.41 (3.71-5.24) | 2.77 (2.28-3.38) | Causal (bidirectional) |
| Hypertension-Osteoarthritis of the hip | 1.97 | 2.63 (2.12-3.26) | 1.42 (1.11-1.83) | 1.52 | 1.31 (1.11-1.55) | 1.17 (0.99-1.39) | Shared determinants (obesity) |
| Cataract-Hypertension | 1.84 | 2.19 (1.72-2.80) | 1.88 (1.45-2.43) | 1.64 | 1.11 (0.94-1.32) | - | Causal (determinant) |
| Diabetes-Obesity, nonmorbid | 1.48 | 5.11 (4.14-6.30) | 3.58 (2.85-4.49) | 1.94 | 3.23 (2.76-3.76) | 2.83 (2.40-3.33) | Causal (determinant) |
| Hypertension-Cardiac rhythm disorders | 2.18 | 3.34 (2.64-4.23) | 2.68 (2.08-3.45) | 1.05 | 1.91 (1.53-2.39) | 1.62 (1.28-2.03) | Causal (determinant) |
| Depression-Low back pain | 1.68 | 3.12 (2.58-3.77) | 1.43 (1.14-1.79) | 1.50 | 2.62 (2.22-3.09) | 1.64 (1.37-1.98) | Causal (bidirectional) |
| Anxiety-Osteoarthritis of other peripheral joints | 1.75 | 3.07 (2.54-3.72) | 1.98 (1.59-2.42) | 1.33 | 2.60 (2.17-3.12) | 1.89 (1.56-2.29) | Causal (bidirectional) |
| Anxiety-Osteoarthritis of the knee | 1.92 | 3.11 (2.57-3.75) | 1.71 (1.36-2.15) | 1.08 | 2.44 (2.04-2.93) | 1.50 (1.22-1.83) | Causal (bidirectional) |
| Osteoarthritis of the hip-Osteoarthritis of other peripheral joints | 1.24 | 3.49 (2.73-4.46) | 1.69 (1.26-2.26) | 1.76 | 3.14 (2.61-3.79) | 2.20 (1.81-2.68) | Strongly related pathological processes |
| Thyroid disorders-Hypertension | 1.57 | 2.37 (1.89-2.97) | 1.79 (1.39-2.31) | 1.39 | 1.70 (1.40-2.05) | 1.49 (1.22-1.81) | Causal (complication) |

Abbreviations. OR: odds ratio; NT: not tested due to a limited number of subjects with the condition; COPD: chronic obstructive pulmonary disease

* Full models include age-sex and all conditions associated independently with both components of the dyad (see Fig. 1)

S7 Table (continued). Weighted frequency of dyads of conditions, strength of associations, and plausible etiological pathways explaining the associations. All dyads with a frequency of ≥ 0.25% in at least one survey sample are considered. Dyads are presented in decreasing order of frequency (mean frequency based on the two surveys).

|  | 1-year time frame (ESPS Survey) | | | | | | Lifetime frame (HSM Survey) | | | | | |  | |
| --- | --- | --- | --- | --- | --- | --- | --- | --- | --- | --- | --- | --- | --- | --- |
| Dyad | Frequency | | Age- and sex-adjusted OR | | Fully adjusted* OR | | Frequency | | Age- and sex-adjusted OR | | Fully adjusted* OR | | Plausible etiological pathway(s) explaining the association | |
| Obesity, nonmorbid-Osteoarthritis of other peripheral joints | 1.21 | 1.94 (1.57-2.39) | | 1.23 (0.96-1.57) | | 1.72 | | 1.36 (1.18-1.58) | | 1.19 (1.03-1.39) | | Causal (determinant) | |  |
| Ear ailments-Osteoarthritis of the knee | 2.07 | 2.80 (2.32-3.67) | | 1.64 (1.34-2.01) | | 0.80 | | 1.65 (1.34-2.04) | | 1.32 (1.10-1.69) | | Shared determinants (mental) | |  |
| Thyroid disorders-Low back pain | 1.13 | 1.92 (1.55-2.39) | | 1.32 (1.03-1.70) | | 1.57 | | 1.91 (1.60-2.27) | | 1.53 (1.07-1.85) | | Causal (complication) | |  |
| Migraine-Hypertension | 1.10 | 1.70 (1.35-2.14) | | 1.11 (0.86-1.45) | | 1.52 | | 1.28 (1.09-1.50) | | 1.07 (0.90-1.28) | | Confounding | |  |
| Anxiety-Ear ailments | 1.94 | 3.64 (3.07-4.37) | | 2.41 (1.97-2.96) | | 0.61 | | 2.30 (1.84-2.87) | | 1.61 (1.26-2.06) | | Causal (bidirectional) | |  |
| Cataract-Low back pain | 1.09 | 2.30 (1.81-2.93) | | 1.25 (0.95-1.64) | | 1.42 | | 1.58 (1.32-1.88) | | 1.37 (1.14-1.64) | | Shared determinants (obesity, smoking) | |  |
| Migraine-Osteoarthritis of other peripheral joints | 0.94 | 2.79 (2.20-3.55) | | 1.61 (1.21-2.15) | | 1.56 | | 2.13 (1.81-2.50) | | 1.48 (1.23-1.77) | | Shared determinants (obesity, mental) | |  |
| Depression-Hypertension | 1.56 | 2.15 (1.71-2.70) | | 1.31 (1.00-1.71) | | 0.89 | | 1.42 (1.18-1.70) | | 1.15 (0.94-1.40) | | Chance | |  |
| Asthma-Low back pain | 0.93 | 2.78 (2.18-3.54) | | 1.67 (1.25-2.22) | | 1.48 | | 1.51 (1.26-1.81) | | 1.38 (1.15-1.67) | | Shared determinants (environmental, smoking, mental) | |  |
| Diabetes-Low back pain | 1.04 | 1.58 (1.26-1.97) | | 1.12 (0.89-1.41) | | 1.35 | | 1.10 (0.93-1.29) | | - | | Confounding | |  |
| Low back pain-Injury sequelae | 0.41 | 2.99 (2.10-4.24) | | 2.58 (1.76-3.78) | | 1.98 | | 2.36 (2.01-2.77) | | 1.73 (1.46-2.05) | | Causal (complication) | |  |
| Cataract-Osteoarthritis of the knee | 1.22 | 3.01 (2.34-3.88) | | 2.11 (1.60-2.78) | | 1.15 | | 1.31 (1.09-1.59) | | 1.14 (0.94-1.39) | | Shared determinants (obesity) | |  |
| Depression-Migraine | 1.19 | 4.72 (3.79-5.88) | | 2.10 (1.63-2.72) | | 1.16 | | 3.86 (3.23-4.63) | | 1.95 (1.59-2.40) | | Causal (bidirectional) | |  |
| Ear ailments-Osteoarthritis of other peripheral joints | 1.45 | 2.06 (1.66-2.56) | | 1.22 (0.96-1.56) | | 0.85 | | 1.47 (1.21-1.80) | | 1.19 (0.97-1.46) | | Chance | |  |
| Cardiac rhythm disorders-Low back pain | 1.25 | 2.29 (1.84-2.86) | | 1.49 (1.17-1.90) | | 1.03 | | 2.04 (1.67-2.49) | | 1.48 (1.20-1.83) | | Associated determinants (obesity, vascular) | |  |
| Migraine-Osteoarthritis of the knee | 1.01 | 3.07 (2.44-3.87) | | 1.61 (1.23-2.11) | | 1.25 | | 2.17 (1.84-2.56) | | 1.59 (1.32-1.90) | | Shared determinants (obesity, mental) | |  |
| Obesity, non morbid-Ear ailments | 1.42 | 2.07 (1.71-2.51) | | 1.47 (1.19-1.81) | | 0.80 | | 1.17 (0.94-1.47) | | - | | Shared determinants (mental) | |  |
| Diabetes-Osteoarthritis of the knee | 1.12 | 2.16 (1.72-2.73) | | 1.23 (0.95-1.58) | | 1.08 | | 1.57 (1.31-1.87) | | 1.20 (1.00-1.45) | | Confounding | |  |
| COPD-Low back pain | 0.79 | 3.51 (2.67-4.61) | | 1.69 (1.20-2.39) | | 1.41 | | 1.82 (1.53-2.16) | | 1.22 (1.00-1.48) | | Shared determinants (smoking) | |  |
| Obesity, non morbid-Anxiety | 1.21 | 1.58 (1.30-1.91) | | 0.88 (0.69-1.13) | | 0.88 | | 1.28 (1.07-1.53) | | 1.05 (0.87-1.27) | | Confounding | |  |
| Low back pain-Osteoporosis | 1.04 | 2.76 (2.15-3.53) | | 2.28 (1.73-3.01) | | 0.97 | | 2.04 (1.65-2.53) | | 1.88 (1.51-2.34) | | Causal (complication) | |  |
| Cataract-Osteoarthritis of other peripheral joints | 0.72 | 1.36 (1.01-1.83) | | 0.95 (0.69-1.31) | | 1.29 | | 1.37 (1.14-1.64) | | 1.22 (1.00-1.48) | | Confounding | |  |
| Migraine-Ear ailments | 1.05 | 3.53 (2.80-4.46) | | 2.00 (1.52-2.64) | | 0.95 | | 2.59 (2.11-3.18) | | 2.06 (1.64-2.58) | | Shared determinants (obesity, mental) | |  |
| Thyroid disorders-Osteoarthritis of other peripheral joints | 0.92 | 2.20 (1.70-2.85) | | 1.68 (1.27-2.23) | | 1.06 | | 1.64 (1.35-2.00) | | 1.40 (1.14-1.71) | | Causal (complication) | |  |
| Hypertension-COPD | 0.80 | 2.13 (1.54-2.93) | | 1.25 ( 0.88-1.78) | | 1.12 | | 1.15 (0.96-1.37) | | - | | Confounding | |  |
| Obesity, nonmorbid-Osteoarthritis of the hip | 1.01 | 2.82 (2.23-3.57) | | 1.45 (1.10-1.90) | | 0.91 | | 1.27 (1.06-1.53) | | 0.94 (0.77-1.14) | | Causal (bidirectional) | |  |
| Depression-Osteoarthritis of other peripheral joints | 0.98 | 2.44 (1.90-3.14) | | 1.26 (0.93-1.70) | | 0.92 | | 2.37 (1.96-2.87) | | 1.56 (1.26-1.93) | | Causal (bidirectional) | |  |
| Peptic ulcer-Low back pain | 0.63 | 3.78 (2.78-5.14) | | 2.51 (1.75-3.60) | | 1.24 | | 2.44 (2.01-2.96) | | 1.65 (1.34-2.01) | | Shared determinants (mental) | |  |

Abbreviations. OR: odds ratio; NT: not tested due to a limited number of subjects with the condition; COPD: chronic obstructive pulmonary disease

* Full models include age-sex and all conditions associated independently with both components of the dyad (see Fig. 1)

S7 Table (continued). Weighted frequency of dyads of conditions, strength of associations, and plausible etiological pathways explaining the associations. All dyads with a frequency of ≥ 0.25% in at least one survey sample are considered. Dyads are presented in decreasing order of frequency (mean frequency based on the two surveys).

|  | 1-year time frame (ESPS Survey) | | | | | | Lifetime frame (HSM Survey) | | |  |  |
| --- | --- | --- | --- | --- | --- | --- | --- | --- | --- | --- | --- |
| Dyad | Frequency | | Age- and sex-adjusted OR | | Fully adjusted* OR | | Frequency | Age- and sex-adjusted OR | Fully adjusted* OR | Plausible etiological pathway(s) explaining the association |  |
| Anxiety-Osteoarthritis of the hip | 1.07 | 3.06 (2.42-3.87) | | 1.37 (1.04-1.81) | | 0.79 | | 2.80 (2.24-3.51) | 1.53 (1.19-1.96) | Causal (bidirectional) |  |
| Depression-Osteoarthritis of the knee | 1.06 | 2.45 (1.93-3.11) | | 1.12 (0.85-1.49) | | 0.79 | | 2.53 (2.11-3.04) | 1.53 (1.24-1.88) | Causal (bidirectional) | |
| Hypertension-Osteoporosis | 1.05 | 1.28 (0.98-1.67) | | - | | 0.79 | | 1.05 (0.84-1.32) | - | Chance | |
| Thyroid disorders-Obesity, nonmorbid | 0.92 | 2.58 (2.04-3.27) | | 1.89 (1.47-2.44) | | 0.92 | | 1.39 (1.13-1.69) | 1.27 (1.04-1.56) | Causal (complication) | |
| Hypertension-Ischemic heart disease | 0.76 | 2.80 (1.93-4.06) | | 1.69 (1.12-2.54) | | 1.08 | | 1.63 (1.33-2.01) | 1.38 (1.12-1.71) | Causal (determinant) | |
| Cardiac rhythm disorders-Osteoarthritis of the knee | 1.19 | 2.39 (1.88-3.04) | | 1.49 (1.16-1.93) | | 0.62 | | 1.72 (1.38-2.14) | 1.25 (0.97-1.60) | Shared determinants (obesity) | |
| Obesity, nonmorbid-Depression | 1.05 | 2.55 (2.05-3.18) | | 2.12 (1.68-2.67) | | 0.71 | | 1.38 (1.13-1.69) | 1.23 (1.00-1.52) | Causal (bidirectional) | |
| Thyroid disorders-Osteoarthritis of the knee | 1.04 | 2.48 (1.93-3.20) | | 1.65 (1.23-2.22) | | 0.72 | | 1.30 (1.06-1.60) | 0.97 (0.78-1.19) | Causal (complication) | |
| Hypertension -Asthma | 0.82 | 2.48 (1.85-3.32) | | 1.79 (1.30-2.48) | | 0.94 | | 1.12 (0.91-1.37) | - | Causal (determinant) | |
| Obesity, nonmorbid-Migraine | 0.71 | 1.38 (1.09-1.75) | | 0.87 (0.66-1.14) | | 1.02 | | 0.85 (0.72-1.00) | - | Confounding | |
| Thyroid disorders-Anxiety | 0.96 | 2.01 (1.59-2.54) | | 1.20 (0.88-1.64) | | 0.73 | | 2.34 (1.86-2.95) | 1.85 (1.45-2.37) | Causal (complication) | |
| Ear ailments-Osteoarthritis of the hip | 1.20 | 2.88 (2.30-3.62) | | 1.37 (1.04-1.80) | | 0.48 | | 1.53 (1.19-1.98) | 1.11 (0.84-1.47) | Shared determinants (mental) | |
| Cataract-Ear ailments | 1.20 | 3.06 (2.39-3.91) | | 2.36 (1.82-3.07) | | 0.47 | | 1.28 (0.87-1.67) | - | Shared determinants (obesity) | |
| Other inflammatory arthritis-Low back pain | 0.23 | NT | | NT | | 1.44 | | 3.20 (2.63-3.90) | 2.33 (1.89-2.88) | Causal (complication) | |
| Diabetes-Osteoarthritis of other peripheral joints | 0.71 | 1.49 (1.13-1.95) | | 1.14 (0.85-1.51) | | 0.95 | | 1.08 (0.90-1.29) | - | Confounding | |
| Depression-Ear ailments | 1.24 | 3.66 (2.92-4.59) | | 1.89 (1.46-2.46) | | 0.40 | | 1.97 (1.54 -2.52) | 1.25 (0.95-1.65) | Causal (bidirectional) | |
| Obesity, nonmorbid-Asthma | 0.63 | 2.60 (1.99-3.40) | | 2.03 (1.53-2.70) | | 0.95 | | 1.30 (1.05-1.60) | 1.23 (0.99-1.52) | Causal (determinant) | |
| Migraine-Osteoarthritis of the hip | 0.61 | 3.70 (2.75-4.99) | | 1.82 (1.27-2.62) | | 0.90 | | 2.54 (2.08-3.11) | 1.68 (1.33-2.11) | Shared determinants (obesity, mental) | |
| COPD-Asthma | 0.50 | 12.40 (8.88-17.33) | | 9.48 (6.57-13.68) | | 1.01 | | 5.35 (4.34-6.59) | 5.21 (4.22-6.42) | Causal (complication) | |
| Osteoarthritis of the knee-Osteoporosis | 0.76 | 1.72 (1.30-2.28) | | 1.01 (0.71-1.43) | | 0.74 | | 1.87 (1.46-2.39) | 1.72 (1.33-2.23) | Causal (complication) | |
| Osteoarthritis of other peripheral joints-Osteoporosis | 0.71 | 1.80 (1.35-2.40) | | 1.31 (0.95-1.80) | | 0.79 | | 1.62 (1.26-2.07) | 1.41 (1.09-1.82) | Causal (complication) | |
| Hypertension-Stroke | 0.82 | 6.41 (4.13-9.93) | | 4.74 (304-7.41) | | 0.66 | | 2.13 (1.69-2.68) | 1.93 (1.54-2.43) | Causal (determinant) | |
| Cardiac rhythm disorders-Osteoarthritis of other peripheral joints | 0.80 | 1.68 (1.27-2.23) | | 1.05 (0.77-1.44) | | 0.68 | | 1.67 (1.32-2.11) | 1.28 (1.00-1.62) | Confounding | |
| Hypertension-Injury sequelae | 0.38 | 2.06 (1.30-3.25) | | 1.60 (0.99-2.60) | | 1.09 | | 1.20 (1.00-1.43) | - | Confounding | |
| Anxiety-Cardiac rhythm disorders | 0.93 | 2.57 (2.00-3.32) | | 1.67 (1.26-2.21) | | 0.52 | | 3.12 (2.44-3.98) | 2.06 (1.55-2.74) | Causal (complication) | |
| COPD-Osteoarthritis of the knee | 0.63 | 3.27 (2.39-4.47) | | 1.82 (1.31-2.51) | | 0.82 | | 1.56 (1.27-1.92) | 1.36 (1.11-1.66) | Shared determinants (environmental) | |
| Ear ailments-Cardiac rhythm disorders | 1.13 | 2.52 (2.00-3.16) | | 1.55 (1.21-1.99) | | 0.31 | | 1.56 (1.17-2.07) | 1.16 (0.86-1.57) | Shared determinants (vascular) | |
| Migraine-Asthma | 0.58 | 3.00 (2.25-3.99) | | 1.70 (1.1-2.40) | | 0.85 | | 1.45 (1.17-1.81) | 1.09 (0.86-1.38) | Causal (determinant) | |

Abbreviations. OR: odds ratio; NT: not tested due to a limited number of subjects with the condition; COPD: chronic obstructive pulmonary disease

* Full models include age-sex and all conditions associated independently with both components of the dyad (see Fig. 1)

S7 Table (continued). Weighted frequency of dyads of conditions, strength of associations, and plausible etiological pathways explaining the associations. All dyads with a frequency of ≥ 0.25% in at least one survey sample are considered. Dyads are presented in decreasing order of frequency (mean frequency based on the two surveys).

|  | 1-year time frame (ESPS Survey) | | | | | | Lifetime frame (HSM Survey) | | | | | |  |  |
| --- | --- | --- | --- | --- | --- | --- | --- | --- | --- | --- | --- | --- | --- | --- |
| Dyad | Frequency | | Age- and sex-adjusted OR | | Fully adjusted* OR | | Frequency | | Age- and sex-adjusted OR | | Fully adjusted* OR | | Plausible etiological pathway(s) explaining the association |  |
| Thyroid disorders-Migraine | 0.61 | 1.99 (1.52-2.61) | | 1.37 (1.00-1.86) | | 0.81 | | | 1.47 (1.19-1.81) | | 1.04 (0.83-1.30) | | Confounding | |
| Migraine-COPD | 0.39 | 4.30 (3.06-6.05) | | 1.75 (1.13-2.71) | | 1.00 | | | 3.28 (2.67-4.03) | | 2.38 (1.89-2.99) | | Shared determinants (asthma) | |
| Obesity, nonmorbid-Cardiac rhythm disorders | 0.83 | 2.07 (1.62-2.64) | | 1.18 (0.90-1.54) | | 0.54 | | 1.26 (1.01-1.59) | | 1.11 (0.88-1.39) | | Confounding | |  |
| Hypertension-Peptic ulcer | 0.57 | 2.31 (1.62-3.30) | | 1.74 (1.20-2.55) | | 0.80 | | 1.33 (1.08-1.64) | | 1.19 (0.97-1.47) | | Shared determinants (mental, smoking) | |  |
| Anxiety-Cataractact | 0.74 | 2.16 (1.62-2.88) | | 1.30 (0.95-1.77) | | 0.61 | | 1.90 (1.51-2.40) | | 1.47 (1.14-1.89) | | Causal (complication) | |  |
| COPD-Osteoarthritis of other peripheral joints | 0.54 | 3.24 (2.31-4.54) | | 1.87 (1.29-2.73) | | 0.80 | | 1.28 (1.06-1.58) | | 0.99 (0.80-1.22) | | Shared determinants (environmental) | |  |
| Diabetes-Ear ailments | 0.94 | 1.80 (1.43-2.26) | | 1.28 (1.00-1.64) | | 0.40 | | 0.97 (0.74-1.27) | | - | | Confounding | |  |
| Migraine-Injury sequelae | 0.15 | NT | | NT | | 1.18 | | 2.90 (3.38-3.52) | | 1.89 (1.52-2.35) | | Causal (complication) | |  |
| Anxiety-Asthma | 0.75 | 3.03 (2.34-3.92) | | 1.67 (1.22-2.28) | | 0.55 | | 1.76 (1.36-2.29) | | 1.40 (1.06-1.85) | | Causal (bidirectional) | |  |
| Thyroid disorders-Ear ailments | 0.75 | 1.87 (1.44-2.44) | | 1.23 (0.92-1.64) | | 0.54 | | 2.15 (1.63-2.84) | | 1.89 (1.42-2.53) | | Causal (complication) | |  |
| Osteoarthritis of the knee-Injury sequelae | 0.32 | 3.33 (2.16-5.13) | | 2.56 (1.60-4.09) | | 0.97 | | 2.32 (1.90-2.82) | | 1.66 (1.34-2.05) | | Causal (complication) | |  |
| Ischemic heart disease-Low back pain | 0.47 | 2.92 (2.08-4.10) | | 1.94 (1.33-2.82) | | 0.80 | | 1.40 (1.15-1.71) | | 1.23 (1.00-1.51) | | Shared determinants (obesity) | |  |
| Osteoarthritis of other peripheral joints-Injury sequelae | 0.22 | NT | | NT | | 1.05 | | 2.12 (1.74-2.58) | | 1.55 (1.25-1.92) | | Causal (complication) | |  |
| Anxiety-COPD | 0.63 | 4.17 (3.12-5.57) | | 1.83 (1.22-2.75) | | 0.63 | | 2.59 (2.08-3.22) | | 1.29 (1.00-1.69) | | Causal (complication) | |  |
| Asthma-Osteoarthritis of the knee | 0.52 | 2.45 (1.79-3.35) | | 1.16 (0.81-1.66) | | 0.74 | | 1.75 (1.39-2.21) | | 1.44 (1.11-1.87) | | Shared determinants (mental) | |  |
| Asthma-Osteoarthritis of other peripheral joints | 0.51 | 2.62 (1.90-3.60) | | 1.65 (1.16-2.35) | | 0.75 | | 1.36 (1.08-1.71) | | 1.19 (0.94-1.51) | | Shared determinants (mental) | |  |
| Hypertension-Peripheral arterial disease | 0.76 | 4.59 (2.99-7.04) | | 3.67 (2.32-5.79) | | 0.48 | | 2.21 (1.65-2.97) | | 1.59 (1.14-2.21) | | Causal (determinant) | |  |
| Diabetes-Cataract | 0.53 | 1.46 (1.06-2.01) | | 1.06 (0.76-1.48) | | 0.71 | | 1.17 (0.95-1.44) | | - | | Confounding | |  |
| Other inflammatory arthritis-Hypertension | 0.37 | 3.49 (2.20-5.51) | | 2.36 (1.35-4.13) | | 0.87 | | 1.45 (1.16-1.81) | | 1.22 (0.89-1.53) | | Shared determinants (obesity) | |  |
| Thyroid disorders-Depression | 0.75 | 2.67 (2.06-3.46) | | 2.18 (1.65-2.89) | | 0.46 | | 1.83 (1.43-2.35) | | 1.20 (0.90-1.61) | | Causal (complication) | |  |
| Diabetes-Anxiety | 0.65 | 1.49 (1.14-1.96) | | 1.12 (0.83-1.49) | | 0.56 | | 1.54 (1.24-1.92) | | 1.22 (0.96-1.55) | | Confounding | |  |
| Obesity, nonmorbid-COPD | 0.46 | 2.48 (1.80-3.42) | | 1.41 (0.99-2.02) | | 0.75 | | 1.18 (0.98-1.41) | | - | | Confounding | |  |
| Diabetes-Osteoarthritis of the hip | 0.56 | 1.72 (1.28-2.31) | | 1.02 (0.74-1.14) | | 0.64 | | 1.29 (1.05-1.57) | | 1.20 (0.98-1.47) | | Confounding | |  |
| Anxiety-Osteoporosis | 0.73 | 2.07 (1.58-2.72) | | 1.34 (0.99-1.83) | | 0.44 | | 2.18 (1.68-2.83) | | 1.39 (1.00-1.93) | | Confounding | |  |
| Obesity, nonmorbid-Cataract | 0.43 | 1.24 (0.89-1.71) | | - | | 0.74 | | 0.94 (0.76-1.16) | | - | | Chance | |  |
| Migraine-Peptic ulcer | 0.33 | 4.66 (3.25-6.70) | | 2.73 ( 1.77-4.20) | | 0.82 | | 3.48 (2.78-4.36) | | 2.13 (1.64-2.76) | | Shared determinants (mental) | |  |
| Hypertension-Heart failure | 0.21 | NT | | NT | | 0.92 | | 1.55 (1.25-1.92) | | 1.17 (0.94-1.46) | | Confounding | |  |
| Low back pain-Urinary incontinence | 0.08 | NT | | NT | | 1.05 | | 2.29 (1.82-2.73) | | 1.62 (1.31-2.00) | | Shared determinants (obesity, mental) | |  |
| Thyroid disorders-Osteoarthritis of the hip | 0.57 | 2.17 (1.58-2.99) | | 1.26 (0.86-1.85) | | 0.55 | | 1.72 (1.34-2.21) | | 1.26 (0.97-1.65) | | Confounding | |  |

Abbreviations. OR: odds ratio; NT: not tested due to a limited number of subjects with the condition; COPD: chronic obstructive pulmonary disease

* Full models include age-sex and all conditions associated independently with both components of the dyad (see Fig. 1)

S7 Table (continued). Weighted frequency of dyads of conditions, strength of associations, and plausible etiological pathways explaining the associations. All dyads with a frequency of ≥ 0.25% in at least one survey sample are considered. Dyads are presented in decreasing order of frequency (mean frequency based on the two surveys).

|  | 1-year time frame (ESPS Survey) | | | | | Lifetime frame (HSM Survey) | | |  |  |
| --- | --- | --- | --- | --- | --- | --- | --- | --- | --- | --- |
| Dyad | Frequency | | Age- and sex-adjusted OR | | Fully adjusted* OR | Frequency | Age- and sex-adjusted OR | Fully adjusted* OR | Plausible etiological pathway(s) explaining the association |  |
| Glaucoma-Hypertension | 0.66 | 1.56 (1.12-2.17) | | 1.15 (0.80-1.67) | | 0.42 | 1.32 (1.00-1.75) | - | Chance | |
| Thyroid disorders-Diabetes | 0.51 | 2.18 (1.58-3.01) | | 1.43 (1.02-2.01) | | 0.57 | 1.70 (1.32-2.18) | 1.33 (1.01-1.75) | Causal (complication) | |
| Cataract-Osteoporosis | 0.57 | 3.03 (2.10-4.36) | | 2.59 (1.76-3.81) | | 0.51 | 1.67 (1.27-2.21) | 1.46 (1.10-1.94) | Shared determinants (smoking) | |
| Obesity, nonmorbid-Injury sequelae | 0.24 | NT | | NT | | 0.84 | 1.11 (0.91-1.34) | - | Chance | |
| Glaucoma-Low back pain | 0.63 | 2.95 (2.15-4.05) | | 2.42 (1.70-3.45) | | 0.44 | 1.71 (1.27-2.29) | 1.47 (1.09-1.99) | Associated determinants (obesity, vascular) | |
| Anxiety-Peptic ulcer | 0.45 | 3.92 (2.82-5.45) | | 2.03 (1.39-2.95) | | 0.62 | 3.84 (2.99-4.93) | 1.98 (1.44-2.71) | Causal (bidirectional) | |
| Other inflammatory arthritis-Osteoarthritis of the knee | 0.17 | NT | | NT | | 0.89 | 3.19 (2.53-4.01) | 2.18 (1.68-2.82) | Causal (complication) | |
| Ear ailments-Asthma | 0.63 | 3.38 (2.50-4.57) | | 2.15 (1.55-2.97) | | 0.42 | 1.43 (1.10-1.86) | 1.28 (0.98-1.67) | Shared determinants (mental) | |
| Hypertension-Myocardial infarction | 0.53 | 2.76 (1.79-4.26) | | 1.72 (1.07-2.76) | | 0.52 | 1.22 (0.93-1.60) | - | Causal (determinant) | |
| Diabetes-Ischemic heart disease | 0.39 | 3.90 (2.60-5.86) | | 2.57 (1.66-3.98) | | 0.66 | 2.32 (1.84-2.94) | 1.89 (1.48-2.41) | Causal (determinant) | |
| Osteoarthritis of the hip-Osteoporosis | 0.57 | 2.66 (1.92-3.70) | | 2.02 (1.43-2.86) | | 0.48 | 1.83 (1.42-2.36) | 1.34 (1.02-1.75) | Causal (complication) | |
| Thyroid disorders-Cardiac rhythm disorders | 0.57 | 2.41 (1.76-3.29) | | 1.80 (1.29-2.51) | | 0.48 | 2.93 (2.19-2.93) | 2.24 (1.65-3.03) | Causal (complication) | |
| Peptic ulcer-Osteoarthritis of the knee | 0.43 | 3.16 (2.21-4.50) | | 1.36 (0.87-2.12) | | 0.61 | 1.92 (1.53-2.41) | 1.23 (0.97-1.57) | Confounding | |
| Hypertension-Urinary incontinence | 0.12 | NT | | NT | | 0.92 | 1.24 (1.03-1.50) | 1.06 (0.88-1.29) | Confounding | |
| Anxiety-Injury sequelae | 0.25 | 2.49 (1.60-3.86) | | 1.25 (0.71-2.20) | | 0.74 | 2.76 (2.22-3.42) | 1.47 (1.16-1.87) | Causal (complication) | |
| Migraine-Cardiac rhythm disorders | 0.39 | 1.93 (1.39-2.70) | | 1.00 (0.67-1.47) | | 0.59 | 2.57 (2.03-3.26) | 1.77 (1.36-2.31) | Shared determinants (obesity) | |
| Depression-COPD | 0.43 | 4.32 (30.7-6.07) | | 2.17 (1.39-3.38) | | 0.54 | 3.17 (2.52-3.97) | 2.22 (1.74-2.84) | Causal (complication) | |
| Peptic ulcer-Osteoarthritis of other peripheral joints | 0.25 | 1.92 (1.26-2.92) | | 1.12 (0.69-1.81) | | 0.70 | 1.93 (1.56-2.39) | 1.40 (1.13-1.75) | Shared determinants (mental) | |
| Ear ailments-Osteoporosis | 0.74 | 2.25 (1.71-2.97) | | 1.50 (1.11-2.03) | | 0.20 | NT | NT | Shared determinants (smoking) | |
| Diabetes-Depression | 0.48 | 1.86 (1.36-2.54) | | 1.47 (1.06-2.02) | | 0.46 | 1.87 (1.46-2.39) | 1.65 (1.28-2.14) | Causal (complication) | |
| Thyroid disorders-Cataract | 0.43 | 1.50 (1.03-2.17) | | 1.12 (0.77-1.64) | | 0.51 | 1.74 (1.34-2.27) | 1.54 (1.18-2.01) | Causal (complication) | |
| Thyroid disorders-Osteoporosis | 0.48 | 1.65 (1.17-2.32) | | 1.57 (1.11-2.23) | | 0.45 | 1.68 (1.25-2.26) | 1.55 (1.14-2.10) | Causal (complication) | |
| Diabetes-COPD | 0.33 | 2.52 (1.70-3.74) | | 1.54 (1.00-2.39) | | 0.59 | 1.45 (1.18-1.78) | 1.18 (0.95-1.46) | Confounding | |
| Obesity, nonmorbid-Ischemic heart disease | 0.34 | 2.78 (1.91-4.04) | | 1.67 (1.08-2.57) | | 0.56 | 1.33 (1.06-1.67) | 1.05 (0.83-1.34) | Causal (determinant) | |
| Migraine-Cataract | 0.30 | 2.32 (1.54-3.51) | | 1.43 (0.91-2.26) | | 0.60 | 2.06 (1.62-2.60) | 1.31 (0.98-1.73) | Confounding | |
| Cataract-COPD | 0.21 | NT | | NT | | 0.67 | 1.94 (1.54-2.45) | 1.74 (1.37-2.20) | Shared determinants (smoking) | |
| Obesity, nonmorbid-Peptic ulcer | 0.27 | 1.76 (1.20-2.57) | | 1.15 (0.75-1.77) | | 0.61 | 1.38 (1.08-1.76) | 1.27 (0.99-1.63) | Confounding | |
| Depression-Asthma | 0.48 | 3.10 (2.30-4.18) | | 1.39 (0.94-2.06) | | 0.39 | 1.58 (1.24-2.02) | 1.05 (0.81-1.36) | Confounding | |
| Diabetes-Migraine | 0.30 | 1.35 (0.94-1.93) | | - | | 0.56 | 1.10 (0.90-1.35) | - | Chance | |

Abbreviations. OR: odds ratio; NT: not tested due to a limited number of subjects with the condition; COPD: chronic obstructive pulmonary disease

* Full models include age-sex and all conditions associated independently with both components of the dyad (see Fig. 1)

S7 Table (continued). Weighted frequency of dyads of conditions, strength of associations, and plausible etiological pathways explaining the associations. All dyads with a frequency of ≥ 0.25% in at least one survey sample are considered. Dyads are presented in decreasing order of frequency (mean frequency based on the two surveys).

|  | 1-year time frame (ESPS Survey) | | | | | | Lifetime frame (HSM Survey) | | | | | |  |  |
| --- | --- | --- | --- | --- | --- | --- | --- | --- | --- | --- | --- | --- | --- | --- |
| Dyad | Frequency | | Age- and sex-adjusted OR | | Fully adjusted* OR | | Frequency | | Age- and sex-adjusted OR | | Fully adjusted* OR | | Plausible etiological pathway(s) explaining the association |  |
| Osteoarthritis of other peripheral joints-Urinary incontinence | 0.08 | NT | | NT | | | 0.78 | | 1.59 (1.29-1.96) | | 1.20 (0.97-1.29) | | Confounding | |
| Asthma-Osteoarthritis of the hip | 0.35 | 3.31 (2.27-4.83) | | 1.47 (1.00-2.17) | | 0.51 | | 2.00 (1.56-2.58) | | 1.65 (1.25-2.17) | | Shared determinants (mental) | |  |
| Ischemic heart disease-Osteoarthritis of other peripheral joints | 0.31 | 2.13 (1.40-3.26) | | 1.53 (1.00-2.35) | | 0.54 | | 1.05 (0.83-1.32) | | - | | Confounding | |  |
| Glaucoma-Osteoarthritis of the knee | 0.5 | 2.32 (1.62-3.32) | | 1.66 (1.11-2.48) | | 0.35 | | 1.87 (1.32-2.66) | | 1.60 (1.11-2.30) | | Associated determinants (obesity, vascular) | |  |
| Peripheral arterial disease-Low back pain | 0.44 | 2.84 (1.96-4.10) | | 2.23 (1.53-3.24) | | 0.41 | | 2.35 (1.77-3.14) | | 1.86 (1.38-2.251) | | Shared determinants (obesity, smoking) | |  |
| Migraine-Osteoporosis | 0.37 | 1.99 (1.39-2.85) | | 1.32 (0.89-1.97) | | 0.48 | | 1.88 (1.45-2.43) | | 1.34 (1.00-1.79) | | Confounding | |  |
| Thyroid disorders-Asthma | 0.32 | 2.10 (1.45-3.04) | | 1.38 (0.93-2.04) | | 0.51 | | 1.67 (1.24-2.26) | | 1.51 (1.12-2.05) | | Causal (complication) | |  |
| Anxiety-Other inflammatory arthritis | 0.19 | NT | | NT | | 0.63 | | 3.59 (2.82-4.57) | | 2.24 (1.74-2.88) | | Causal (complication) | |  |
| Depression-Injury sequelae | 0.23 | NT | | NT | | 0.58 | | 2.82 (2.23-3.58) | | 1.53 (1.18-1.98) | | Causal (complication) | |  |
| Other inflammatory arthritis-Obesity-non morbid | 0.20 | NT | | NT | | 0.60 | | 1.34 (1.04-1.73) | | 1.15 (0.88-1.50) | | Confounding | |  |
| Inflammatory bowel diseases-Low back pain | 0.75 | 2.90 (2.24-3.76) | | 2.17 (1.65-2.86) | | 0.05 | | NT | | NT | | Shared determinants (smoking, mental) | |  |
| Diabetes-Asthma | 0.30 | 2.56 (1.73-3.78) | | 1.87 (1.24-2.82) | | 0.49 | | 1.45 (1.12-1.89) | | 1.45 (1.12-1.89) | | Shared determinants (inflammation) | |  |
| Ear ailments-Peptic ulcer | 0.43 | 3.26 (2.21-4.81) | | 2.05 (1.33-2.16) | | 0.35 | | 1.75 (1.26-2.43) | | 1.29 (0.91-1.82) | | Shared determinants (mental) | |  |
| Peptic ulcer-Osteoarthritis of the hip | 0.29 | 4.14 (2.74-6.25) | | 2.61 (1.61-4.21) | | 0.48 | | 2.50 (1.90-2.27) | | 1.60 (1.20-2.12) | | Associated determinants (obesity, mental) | |  |
| Heart failure-Low back pain | 0.09 | NT | | NT | | 0.68 | | 1.39 (1.14-1.69) | | 1.05 (0.84-1.31) | | Confounding | |  |
| Cardiac rhythm disorders-Asthma | 0.38 | 3.81 (2.62-5.56) | | 2.65 (1.81-3.88) | | 0.38 | | 2.31 (1.67-3.19) | | 1.79 (1.27-2.52) | | Causal (complication) | |  |
| Cataract-Glaucoma | 0.35 | 3.38 (2.19-5.22) | | 2.92 (1.86-5.58) | | 0.41 | | 4.03 (2.88-5.64) | | 3.79 (2.69-5.34) | | Causal (complication) | |  |
| Ear ailments-Injury sequelae | 0.19 | NT | | NT | | 0.57 | | 1.81 (1.41-2.31) | | 1.55 (1.20-2.00) | | Causal (complication) | |  |
| Osteoarthritis of the hip-Injury sequelae | 0.20 | NT | | NT | | 0.56 | | 1.98 (1.55-2.51) | | 1.14 (0.89-1.47) | | Confounding | |  |
| Other inflammatory arthritis-Osteoarthritis of other peripheral joints | 0.13 | NT | | NT | | 0.63 | | 1.47 (1.15-1.89) | | 0.95 (0.75-1.21) | | Confounding | |  |
| Cardiac rhythm disorders-COPD | 0.26 | 1.87 (1.23-2.84) | | 1.24 (0.78-1.95) | | 0.49 | | 2.90 (2.28-3.70) | | 2.00 (1.53-2.61) | | Shared determinants (smoking) | |  |
| COPD-Injury sequelae | 0.11 | NT | | NT | | 0.64 | | 2.41 (1.92-2.03) | | 1.63 (1.26-2.12) | | Causal (complication) | |  |
| Cataract-Urinary incontinence | 0.05 | NT | | NT | | 0.69 | | 2.31 (1.83-2.93) | | 1.94 (1.52-2.47) | | Shared determinants (obesity) | |  |
| Ischemic heart disease-Cardiac rhythm disorders | 0.39 | 4.12 (2.71-6.24) | | 3.31 (2.14-5.14) | | 0.34 | | 2.43 (1.86-3.18) | | 1.42 (1.04-1.92) | | Causal (complication) | |  |
| Cardiac rhythm disorders-Heart failure | 0.17 | NT | | NT | | 0.54 | | 5.97 (4.61-7.74) | | 4.97 (3.78-6.54) | | Causal (complication) | |  |
| Other inflammatory arthritis-Osteoarthritis of the hip | 0.07 | NT | | NT | | 0.61 | | 3.28 (2.53-4.25) | | 2.35 (1.82-3.03) | | Causal (complication) | |  |
| COPD-Peptic ulcer | 0.16 | NT | | NT | | 0.52 | | 3.11 (2.44-3.97) | | 2.15 (1.61-2.85) | | Shared determinants (smoking) | |  |
| Diabetes-Myocardial infarction | 0.29 | 4.03 (2.56-6.35) | | 3.05 (1.90-4.91) | | 0.38 | | 2.13 (1.62-2.80) | | 1.64 (1.21-2.22) | | Causal (complication) | |  |

Abbreviations. OR: odds ratio; NT: not tested due to a limited number of subjects with the condition; COPD: chronic obstructive pulmonary disease

* Full models include age-sex and all conditions associated independently with both components of the dyad (see Fig. 1)

S7 Table (continued). Weighted frequency of dyads of conditions, strength of associations, and plausible etiological pathways explaining the associations. All dyads with a frequency of ≥ 0.25% in at least one survey sample are considered. Dyads are presented in decreasing order of frequency (mean frequency based on the two surveys).

|  | 1-year time frame (ESPS Survey) | | | | | | Lifetime frame (HSM Survey) | | | | | |  |
| --- | --- | --- | --- | --- | --- | --- | --- | --- | --- | --- | --- | --- | --- |
| Dyad | Frequency | | Age- and sex-adjusted OR | | Fully adjusted* OR | | Frequency | | Age- and sex-adjusted OR | | Fully adjusted* OR | | Plausible etiological pathway(s) explaining the association |
| Myocardial infarction-Low back pain | 0.26 | 1.69 (1.07-2.66) | | 1.46 (0.91-2.34) | | 0.41 | | 1.10 (0.85-1.43) | | - | | Confounding | |
| Diabetes-Injury sequelae | 0.15 | NT | | NT | | 0.52 | | 1.28 (1.01-1.63) | | 1.20 (0.94-1.52) | | Confounding | |
| Obesity, morbid-Hypertension | 0.33 | 11.35 (6.51-19.80) | | 6.91 (3.56-13.41) | | 0.33 | | 4.27 (2.83-6.42) | | 3.20 (2.10-4.87) | | Causal (determinant) | |
| Asthma-Injury sequelae | 0.07 | NT | | NT | | 0.59 | | 1.72 (1.33-2.21) | | 1.36 (1.05-1.76) | | Causal (complication) | |
| Myocardial infarction-Ischemic heart disease | 0.16 | NT | | NT | | 0.50 | | 5.97 (4.47-8.04) | | 4.29 (3.12-5.88) | | Strongly related pathological processes | |
| Obesity, nonmorbid-Heart failure | 0.12 | NT | | NT | | 0.53 | | 1.51 (1.21-1.88) | | 1.25 (0.99-1.59) | | Confounding | |
| Rheumatoid arthritis-Low back pain | 0.06 | NT | | NT | | 0.58 | | 1.96 (1.52-2.51) | | 1.50 (1.15-1.95) | | Causal (complication) | |
| Ischemic heart disease-COPD | 0.13 | NT | | NT | | 0.50 | | 2.50 (1.96-3.19) | | 1.89 (1.45-2.47) | | Shared determinants (smoking) | |
| Anxiety-Urinary incontinence | 0.08 | NT | | NT | | 0.55 | | 3.32 (2.58-4.26) | | 1.92 (1.44-2.56) | | Causal (bidirectional) | |
| Peptic ulcer-Injury sequelae | 0.08 | NT | | NT | | 0.55 | | 2.87 (2.22-3.72) | | 1.91 (1.44-2.54) | | Shared determinants (mental) | |
| Obesity, nonmorbid-Urinary incontinence | 0.07 | NT | | NT | | 0.55 | | 1.34 (1.08-1.66) | | 1.33 (1.07-1.65) | | Causal (complication) | |
| Cataract-Injury sequelae | 0.07 | NT | | NT | | 0.55 | | 2. 23 (1.72-2.89) | | 1.79 (1.37-2.33) | | Causal (complication) | |
| Migraine-Urinary incontinence | 0.02 | NT | | NT | | 0.60 | | 2.91 (2.30-3.68) | | 1.66 (1.26-2.19) | | Shared determinants (obesity, mental) | |
| Ischemic heart disease-Heart failure | 0.06 | NT | | NT | | 0.55 | | 4.49 (3.53-5.71) | | 2.94 (2.26-3.85) | | Causal (complication) | |
| Anxiety-Inflammatory bowel diseases | 0.60 | 3.08 (2.28-4.16) | | 2.13 (1.50-3.02) | | 0.01 | | NT | | NT | | Causal (complication) | |
| Stroke-Low back pain | 0.26 | 1.34 (0.88-2.05) | | - | | 0.35 | | 0.98 (0.77-1.25) | | - | | Chance | |
| Hypertension-Rheumatoid arthritis | 0.14 | NT | | NT | | 0.46 | | 1.15 (0.88-1.52) | | - | | Chance | |
| Ear ailments-Stroke | 0.44 | 3.58 (2.43-5.28) | | 2.40 (1.59-3.61) | | 0.12 | | NT | | NT | | Shared determinants (vascular) | |
| Diabetes-Peripheral arterial disease | 0.3 | 3.34 (2.16-5.18) | | 1.81 (1.13-2.89) | | 0.26 | | 2.32 (1.76-3.06) | | 1.49 (1.11-20.3) | | Causal (complication) | |
| Heart failure-COPD | 0.02 | NT | | NT | | 0.54 | | 3.56 (2.81-4.50) | | 2.31 (1.80-2.97) | | Shared determinants (smoking) | |
| Hypertension-Inflammatory bowel diseases | 0.53 | 1.77 (1.25-2.50) | | 1.41 (0.99-2.00) | | 0.02 | | NT | | NT | | Confounding | |
| Prostate cancer-Hypertension | 0.40 | 2.41 (1.55-3.75) | | 2.12 (1.35-3.34) | | 0.14 | | NT | | NT | | Causal (determinant) | |
| Glaucoma-Ear ailments | 0.40 | 1.96 (1.38-2.78) | | 1.33 (0.88-1.99) | | 0.13 | | NT | | NT | | Confounding | |
| Cataract-Asthma | 0.20 | NT | | NT | | 0.33 | | 1.44 (1.10-1.89) | | 1.15 (0.87-1.53) | | Confounding | |
| Cardiac rhythm disorders-Stroke | 0.34 | 4.58 (2.92-7.19) | | 2.85 (1.82-4.48) | | 0.18 | | NT | | NT | | Causal (determinant) | |
| COPD-Urinary incontinence | 0.04 | NT | | NT | | 0.48 | | 2.73 (2.17-3.43) | | 1.80 (1.39-2.34) | | Causal (complication) | |
| Thyroid disorders-Injury sequelae | 0.13 | NT | | NT | | 0.38 | | 1.47 (1.10-1.95) | | 1.14 (0.84-1.53) | | Confounding | |
| Other cancer-Hypertension | 0.27 | 4.92 (2.55-9.48) | | 4.58 (2.34-8.93) | | 0.23 | | NT | | NT | | Shared determinants (behavioral) | |
| Diabetes-Stroke | 0.16 | NT | | NT | | 0.33 | | 1.88 (1.41-2.51) | | 1.49 (1.12-2.00) | | Causal (determinant) | |

Abbreviations. OR: odds ratio; NT: not tested due to a limited number of subjects with the condition; COPD: chronic obstructive pulmonary disease

* Full models include age-sex and all conditions associated independently with both components of the dyad (see Fig. 1)

S7 Table (continued). Weighted frequency of dyads of conditions, strength of associations, and plausible etiological pathways explaining the associations. All dyads with a frequency of ≥ 0.25% in at least one survey sample are considered. Dyads are presented in decreasing order of frequency (mean frequency based on the two surveys).

|  | 1-year time frame (ESPS Survey) | | | | | | Lifetime frame (HSM Survey) | | | | | |  |
| --- | --- | --- | --- | --- | --- | --- | --- | --- | --- | --- | --- | --- | --- |
| Dyad | Frequency | | Age- and sex-adjusted OR | | Fully adjusted* OR | | Frequency | | Age- and sex-adjusted OR | | Fully adjusted* OR | | Plausible etiological pathway(s) explaining the association |
| Migraine-Inflammatory bowel diseases | 0.46 | 2.99 (2.17-4.11) | | 1.91 (1.32-2.76) | | 0.02 | | NT | | NT | | Shared determinants (mental) | |
| Obesity, nonmorbid-Osteoporosis | 0.21 | NT | | NT | | 0.27 | | 0.56 (0.43-0.73) | | 0.55 (0.42-0.73) | | Causal (protective determinant) | |
| Ischemic heart disease-Peripheral arterial disease | 0.16 | NT | | NT | | 0.32 | | 5.86 (4.24-8.09) | | 3.86 (2.69-5.52) | | Strongly related pathological processes | |
| Other inflammatory arthritis-Thyroid disordersoid disorders | 0.05 | NT | | NT | | 0.42 | | 2.03 (1.53-2.68) | | 1.62 (1.21-2.16) | | Shared determinants (genetics) | |
| Depression-Urinary incontinence | 0.05 | NT | | NT | | 0.42 | | 3.57 (2.80-4.56) | | 1.85 (1.38-2.47) | | Causal (bidirectional) | |
| Diabetes-Obesity, morbid | 0.17 | NT | | NT | | 0.29 | | 10.50 (7.18-15.38) | | 8.33 (5.68-12.22) | | Causal (determinant) | |
| Migraine-Ischemic heart disease | 0.11 | NT | | NT | | 0.35 | | 1.90 (1.45-2.48) | | 1.33 (1.00-1.78) | | Confounding | |
| Inflammatory bowel diseases-Osteoarthritis of other peripheral joints | 0.43 | 2.89 (2.05-4.07) | | 1.88 (1.33-2.64) | | 0.02 | | NT | | NT | | Shared determinants (mental) | |
| Myocardial infarction-Heart failure | 0.06 | NT | | NT | | 0.37 | | 4.84 (3.59-6.53) | | 3.08 (2.22-4.28) | | Causal (determinant) | |
| Inflammatory bowel diseases-Osteoarthritis of the knee | 0.41 | 2.45 (1.73-3.46) | | 1.54 (1.08-2.21) | | 0.02 | | NT | | NT | | Shared determinants (mental) | |
| Ear ailments-Inflammatory bowel diseases | 0.39 | 2.39 (1.66-3.44) | | 1.52 (1.05-2.22) | | 0.02 | | NT | | NT | | Associated determinants (smoking, vascular) | |
| Migraine-Stroke | 0.11 | NT | | NT | | 0.28 | | 2.55 (1.95-3.35) | | 2.32 (1.76-3.05) | | Causal (determinant) | |
| Urinary incontinence-Injury sequelae | 0.02 | NT | | NT | | 0.37 | | 2.49 (1.90-3.26) | | 1.61 (1.20-2.15) | | Causal (complication) | |
| Prostate cancer-Low back pain | 0.26 | 2.60 (1.68-4.02) | | 2.28 (1.46-3.56) | | 0.12 | | NT | | NT | | Causal (complication) | |
| Osteoporosis-Injury sequelae | 0.08 | NT | | NT | | 0.30 | | 2.26 (1.67-3.07) | | 1.89 (1.39-2.57) | | Causal (complication) | |
| Obesity, nonmorbid-Inflammatory bowel diseases | 0.35 | 1.67 (1.19-2.35) | | 1.40 (0.99-1.98) | | 0.03 | | NT | | NT | | Confounding | |
| Anxiety-Rheumatoid arthritis | 0.07 | NT | | NT | | 0.30 | | 2.85 (2.08-3.89) | | 2.47 (1.80-3.39) | | Causal (complication) | |
| Heart failure-Urinary incontinence | 0.01 | NT | | NT | | 0.36 | | 2.89 (2.20-3.79) | | 2.36 (1.77-3.15) | | Causal (complication) | |
| Thyroid disorders-Heart failure | 0.04 | NT | | NT | | 0.31 | | 2.13 (1.56-2.92) | | 1.73 (1.24-2.41) | | Causal (complication) | |
| Ischemic heart disease-Osteoporosis | 0.09 | NT | | NT | | 0.26 | | 2.25 (1.60-3.16) | | 2.44 (1.73-2.45) | | Shared determinants (smoking) | |
| Peptic ulcer-Urinary incontinence | 0.01 | NT | | NT | | 0.32 | | 2.91 (2.20-3.86) | | 1.86 (1.37-2.54) | | Shared determinants (obesity, mental) | |
| Heart failure-Peripheral arterial disease | 0.06 | NT | | NT | | 0.26 | | 5.17 (3.58-7.48) | | 2.07 (1.33-3.20) | | Causal (complication) | |
| Depression-Inflammatory bowel diseases | 0.26 | 1.91 (1.28-2.86) | | 0.86 (0.55-1.37) | | 0.01 | | NT | | NT | | Confounding | |
| Other inflammatory arthritis-Rheumatoid arthritis | 0.00 | NT | | NT | | 0.26 | | 3.69 (2.70-5.04) | | 2.74 (2.01-3.75) | | Strongly related pathological processes | |

Abbreviations. OR: odds ratio; NT: not tested due to a limited number of subjects with the condition; COPD: chronic obstructive pulmonary disease

* Full models include age-sex and all conditions associated independently with both components of the dyad (see Fig. 1)
