# Supplementary material for: Estimating and characterizing the burden of multimorbidity in the community: A comprehensive multistep analysis of two large nationwide representative surveys in France
Source: PLoS Med. 2021 Apr 26;18(4):e1003584. doi: 10.1371/journal.pmed.1003584 (PMC8109815; doi:10.1371/journal.pmed.1003584)
Supplement: S8 Table — (DOCX) [file pmed.1003584.s009.docx]

S8 Table. Weighted frequency of triads and tetrads of conditions. All triads and tetrads with a frequency of ≥ 0.50% in at least one survey sample are considered. Triads and tetrads are presented in decreasing order of frequency (mean frequency based on the two surveys).
