# Supplementary material for: Estimating and characterizing the burden of multimorbidity in the community: A comprehensive multistep analysis of two large nationwide representative surveys in France
Source: PLoS Med. 2021 Apr 26;18(4):e1003584. doi: 10.1371/journal.pmed.1003584 (PMC8109815; doi:10.1371/journal.pmed.1003584)
Supplement: S9 Table — (DOCX) [file pmed.1003584.s010.docx]

S9 Table. Impact of associated conditions (dyads) on activity limitations and perceived health in the ESPS and HSM surveys. Figures represent weighted percentages of subjects presenting limitations or deteriorated perceived health at the indicated threshold. All dyads with a frequency of ≥ 0.50% in at least one survey sample are considered. Dyads are presented in decreasing order of frequency (mean frequency based on the two surveys). Impacts in the top 20 of the rankings for each indicator are highlighted in bold.

Abbreviations
COPD: chronic obstructive pulmonary disease; GALI: Global Activity Limitation Indicator; SRH: Self-Reported Health indicator; ADL: limitation in activity of daily living; IADL: limitation in instrumental activity of daily living: NT: not tested due to the limited number of subjects with the condition

S9 Table (continued). Impact of associated conditions (dyads) on activity limitations and perceived health in the ESPS and HSM surveys. Figures represent weighted percentages of subjects presenting limitations or deteriorated perceived health at the indicated threshold. All dyads with a frequency of ≥ 0.50% in at least one survey sample are considered. Dyads are presented in decreasing order of frequency (mean frequency based on the two surveys). Impacts in the top 20 of the rankings for each indicator are highlighted in bold.

Abbreviations
COPD: chronic obstructive pulmonary disease; GALI: Global Activity Limitation Indicator; SRH: Self-Reported Health indicator; ADL: limitation in activity of daily living; IADL: limitation in instrumental activity of daily living: NT: not tested due to the limited number of subjects with the condition
