# Supplementary material for: Estimating and characterizing the burden of multimorbidity in the community: A comprehensive multistep analysis of two large nationwide representative surveys in France
Source: PLoS Med. 2021 Apr 26;18(4):e1003584. doi: 10.1371/journal.pmed.1003584 (PMC8109815; doi:10.1371/journal.pmed.1003584)
Supplement: S11 Table — (DOCX) [file pmed.1003584.s012.docx]

S11 Table. Joint effects of dyads of associated conditions on activity limitations (severely limited in GALI, limited in ≥ 3 ADLs or ≥ 2 IADLs, new limitation) and perceived health (bad or very bad SRH, new health deterioration) in the ESPS and HSM surveys when the conditions were retained in the final models presented in Table 1. Joint effects are evaluated by the ratio of odds ratios (RoOR) and the relative excess risk due to interaction (RERI) in the logistic models, including the conditions, age, sex, and all conditions independently associated with the indicator under study. Only dyads with a frequency of ≥ 0.25% in at least one survey sample are considered. Dyads are presented in decreasing order of frequency (mean frequency based on the two surveys). Only couple of conditions that were independently associated with a given heath status measure were considered for interaction analyses.

Abbreviations
NT: not tested due to the insufficient sample size; GALI: Global Activity Limitation Indicator; SRH: Self-Reported Health indicator

S11 Table (continued). Joint effects of dyads of associated conditions on activity limitations (severely limited in GALI, limited in ≥ 3 ADLs or ≥ 2 IADLs, new limitation) and perceived health (bad or very bad SRH, new health deterioration) in the ESPS and HSM surveys when the conditions were retained in the final models presented in Table 1. Joint effects are evaluated by the ratio of odds ratios (RoOR) and the relative excess risk due to interaction (RERI) in the logistic models, including the conditions, age, sex, and all conditions independently associated with the indicator under study. Only dyads with a frequency of ≥ 0.25% in at least one survey sample are considered. Dyads are presented in decreasing order of frequency (mean frequency based on the two surveys).

Abbreviations
NT: not tested due to the insufficient sample size; GALI: Global Activity Limitation Indicator; SRH: Self-Reported Health indicator
