# Supplementary material for: Estimating and characterizing the burden of multimorbidity in the community: A comprehensive multistep analysis of two large nationwide representative surveys in France
Source: PLoS Med. 2021 Apr 26;18(4):e1003584. doi: 10.1371/journal.pmed.1003584 (PMC8109815; doi:10.1371/journal.pmed.1003584)
Supplement: S12 Table — (DOCX) [file pmed.1003584.s013.docx]

S12 Table. Joint effects of two-by-two associated conditions within triads on activity limitations and perceived health in the ESPS and HSM surveys when the conditions were retained in the final models presented in Table 1. Joint effects are evaluated on both additive and multiplicative scales by the ratio of odds ratios (RoOR) and the relative excess risk due to interaction (RERI) in the logistic models, including the conditions, age, sex, and all conditions independently associated with the indicator under study. All triads with a frequency of ≥ 0.50% in at least one survey sample are considered. Triads are presented in decreasing order of frequency (mean frequency based on the two surveys). Only triplets of conditions that were independently associated with a given heath status measure were considered for interaction analyses.

Abbreviations
GALI: Global Activity Limitation Indicator; SRH: Self-Reported Health indicator
